# Leveraging Interfacial Electric Field for Smart Modulation of Electrode Surface in Nitrate to Ammonia Conversion

**DOI:** 10.1002/advs.202410763

**Published:** 2024-12-02

**Authors:** Kouer Zhang, Yifan Xu, Fatang Liu, Qing Wang, Xiaohong Zou, Mingcong Tang, Michael K.H. Leung, Zhimin Ao, Xunhua Zhao, Xiao Zhang, Liang An

**Affiliations:** ^1^ Department of Mechanical Engineering The Hong Kong Polytechnic University Hung Hom Kowloon Hong Kong SAR 999077 China; ^2^ Ability R&D Energy Research Centre School of Energy and Environment City University of Hong Kong Kowloon Hong Kong SAR 999077 China; ^3^ College of Chemistry and Chemical Engineering Northeast Petroleum University Daqing 163318 China; ^4^ State Key Laboratory of Marine Pollution City University of Hong Kong Kowloon Hong Kong SAR China; ^5^ Advanced Interdisciplinary Institute of Environment and Ecology Guangdong Provincial Key Laboratory of Wastewater Information Analysis and Early Warning Beijing Normal University Zhuhai 519087 China; ^6^ Key Laboratory of Quantum Materials and Devices of Ministry of Education School of Physics Southeast University Nanjing 211189 China; ^7^ Research Institute for Smart Energy The Hong Kong Polytechnic University Hung Hom Kowloon Hong Kong SAR 999077 China

**Keywords:** electrochemistry, environmental chemistry, interfacial electric field, nitrate reduction

## Abstract

The efficiency of nitrate reduction reaction (NO_3_RR) at low nitrate concentration is predominantly hindered by the poor affinity of nitrate ions and competitive hydrogen evolution reaction (HER), particularly in neutral and acidic media. Here, an innovative strategy to leverage the interfacial electric field (IEF) is introduced to boost the NO_3_RR performance. By in situ constructing tannic acid‐metal ion (TA‐M^2+^) crosslinked structure on the electrode surface, the TA‐M^2+^‐CuO NW/Cu foam sample exhibits an exceptional Faraday efficiency of 99.4% at −0.2 V versus reversible hydrogen electrode (RHE) and 83.9% at 0.0 V versus RHE under neutral and acidic conditions, respectively. The computational studies unveil that the TA‐Cu^2+^ complex on the CuO (111) plane induces the increasing concentration of nitrate at the interface, accelerating NO_3_RR kinetics over HER via the IEF effect. This interfacial modulation strategy also contributes the enhanced ammonia production performance when it is employed on commercial electrode materials and flow reactors, exhibiting great potential in practical application. Overall, combined results illustrated multiple merits of the IEF effect, paving the way for future commercialization of NO_3_RR in the ammonia production industry.

## Graphical abstract

A tannic acid‐metal ion (TA‐M^2+^) crosslinked structure is developed to enhance nitrate reduction reaction efficiency. This innovative approach significantly boosts Faraday efficiency by improving nitrate ion affinity and reducing competitive hydrogen evolution. The TA‐M^2+^‐CuO NW/Cu foam structure shows remarkable performance, enhancing ammonia production and demonstrating great potential for practical applications and future commercialization.

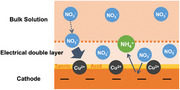

## 1 Introduction

Nitrate reduction reaction (NO_3_RR) is a pivotal green ammonia production pathway while promising for alleviating the nitrate pollution‐induced.^[^ ^1^ ^]^ This electrochemical conversion could reduce 1–2% of global energy consumption and carbon emission,^[^ ^2^ ^]^ superior to the conventional Harbor‐Bosch method. Despite notable performances in alkaline conditions have been achieved, NO_3_RR is still impeded by low selectivity and ammonia yield rates in neutral and acidic electrolytes. This is partly due to a decrease in interfacial nucleophilicity and poor interfacial affinity for nitrate ions at concentrations below 0.1 m.^[^ ^3^ ^]^ During the electrochemical reduction, the negative bias applied to the electrode causes electrostatic repulsion, which hinders the mass transfer of nitrate ions causing inadequate local concentration.^[^ ^4^ ^]^ Meanwhile, the competitive hydrogen evolution reaction (HER) is intensified under neutral and acidic conditions. Hence, formulating a strategy to address the mass‐transfer limitation at the interface and suppress competitive reactions is essential for the effective electroreduction of low‐concentration nitrate to ammonia.^[^ ^5^ ^]^

The interfacial electric field (IEF) effect refers to the localized electric field between the electrode and electrolyte, of which the specific ion concentration at the interfacial region could be increased.^[^ ^6^ ^]^ This strategy has been employed in electrochemical synthesis, especially in processes that yield multiple products or by‐products.^[^ ^7^ ^]^ Cation‐induced IEF, originating from the interaction between positive‐charged cations and negative‐biased electrodes, or from the specific adsorption at the electrode–electrolyte interface, has been regarded as an essential driving force of electrochemical reactions.^[^ ^8^ ^]^ These cations could modulate the IEF via “shielding” the hydronium ions to decrease its local concentration in the electric double layer (EDL), thus suppressing the competitive HER.^[^ ^6^, ^9^ ^]^ Moreover, the cation‐induced IEF effect could enhance the kinetics and thermodynamics of reactions by modulating the adsorption/desorption of electrolyte ions and key intermediates.^[^ ^10^ ^]^ Recently, there already have been some pioneering works focused on electric field and interface modulation, aiming for local nitrate accumulation.^[^ ^3^, ^11^ ^]^ The majority of these studies have primarily focused on either the creation of built‐in electric fields through catalyst design or the cation effects induced by specific adsorption. However, these methods are constrained by the inherently weak and transient nature of the electric fields produced.

In addition to the above methods, the formation of organo–metallic complexes on the electrode surface has emerged as a promising alternative for designing IEF, generating a more powerful and stable IEF effect in comparison.^[^ ^7a^ ^]^ Herein, we demonstrate a novel IEF strategy employing Tannic acid (TA) and a series of metal ions (Cu^2+^, Fe^2+^, Co^2+^) to in situ form the TA‐M^2+^ crosslink structure on the electrode surface. The TA‐Cu^2+^‐CuO NW/Cu foam is prepared through the TA‐Cu^2+^ chelating process as the main sample. This unique structure helps generate stabilized cationic ions at the electrode‐electrolyte interface, beneficial for accumulating nitrate ions through favorable electrostatic interactions, and thus presents prominent enhancement on NO_3_RR selectivity compared to the blank‐CuO NW/Cu foam. The highest ammonia Faraday efficiency (FE) could reach 99.39% at −0.2 V versus RHE and the highest ammonia yield rate could reach 0.99 mmol h^−1^ cm^−2^ at −0.5 V versus RHE in low concentrated nitrate electrolyte (0.5 m K_2_SO_4_ + 2000 ppm KNO_3_). The experiments and computational simulations suggest that modifying the electrode surface with TA‐Cu^2+^ leads to a 1.5‐fold increase in the concentration of nitrate ions at a distance of 1.2 nm from the electrode. Meanwhile, the protons adsorption and reduction are hindered, thereby mitigating the competitive HER. The efficacy of this strategy is demonstrated in the flow reactor, achieving long‐term stability of 100 h with a high FE exceeding 90%. The successful application of this IEF approach to commercial electrode materials underscores its potential for enhancing the selectivity of nitrate‐to‐ammonia conversion, offering a promising avenue for its commercial implementation.

## 2 Results and Discussions

### 2.1 Forming the TA‐M^2±^ Crosslink Organo–Metallic Layer

**Figure 1:**
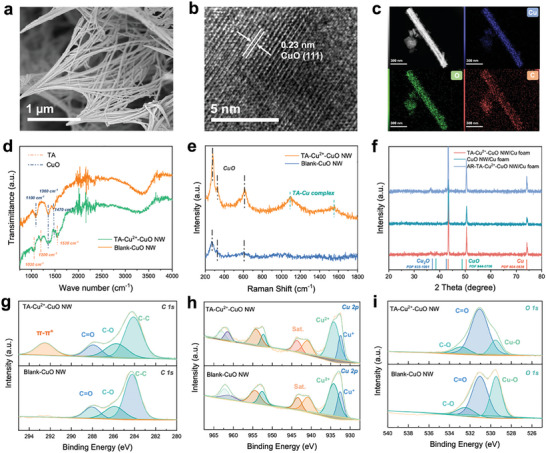
Morphology and structural characterization of TA‐Cu^2+^‐CuO NW. a) SEM image, and b) HRTEM image of TA‐Cu^2+^‐CuO NW. c) The corresponding EDS mapping of Cu, O, and C elements. d) FTIR spectra, e) Raman spectra, and f) XRD patterns of TA‐Cu^2+^‐CuO NW. g, h, i) XPS spectra of C 1s, Cu 2p, and O 1s of TA‐Cu^2+^‐CuO NW/Cu foam and Blank‐CuO NW/Cu foam.

The TA‐M^2+^ crosslink organo‐metallic layer is built on the CuO nanowires structure through a single‐step green approach. First, the CuO NW/Cu foam is synthesized through in situ growth and oxidation. Then, TA and metal ions are simply mixed in deionized water under the pH value of 8.0 as formerly reported.^[^ ^12^ ^]^ In this process, the metal ions accelerate the oxidation of TA catechol groups while the alkaline pH value promotes deprotonation to ensure the self‐assembly of the TA‐M^2+^ crosslink structure.^[^ ^13^ ^]^ In the previous report, under different pH conditions, the coordinating state for TA and metal ions would change correspondingly as shown in Figure S1 (Supporting Information) and tris‐complex is regarded as prominent in this work.^[^ ^14^ ^]^ The synthesis conditions are screened based on electrochemical performance (Figure S2, Supporting Information). Among the synthesized TA‐M^2+^‐CuO NW/Cu foam samples, we mainly focus on TA‐Cu^2+^‐CuO NW/Cu foam. The CuO NW has a diameter of ≈100 nm and no apparent changes appeared after the TA‐Cu^2+^ treatment according to SEM results (**Figure** **1**a; Figures S3, and S4, Supporting Information). To reveal the source of the Cu element in the SEM mappings, the elemental distribution TA‐M^2+^‐CuO NW/Cu foam is also analyzed. The result indicates that the external metal ions are indeed immobilized on the electrode surface by TA‐coordinating (Figures S5 and S6, Supporting Information). The elemental analysis of metallic components for TA‐M^2+^‐CuO NW/Cu foam are presented in Tables S1–S3 (Supporting Information). TEM images further present the morphology of the nanowire structure and the thin layer of the TA‐Cu^2+^ complex which is ≈2 nm could be observed in bright‐field HRTEM (Figure S7, Supporting Information). For the trunk section of the nanowires, CuO (111) could be clearly observed as shown in Figure 1b while the EDS mapping of TA‐Cu^2+^‐CuO NW/Cu foam is shown in Figure 1c. Fourier transform infrared spectrum (FTIR) is applied to validate the presence of TA‐Cu^2+^ coordination on the material surface(Figure 1d). The peaks at 1100, 1160, and 1470 cm^−1^ could be attributed to the CuO, which appear in both the two samples. Compared with the blank CuO NW, obvious peaks at 1030, 1200, and 1530 cm^−1^ could be observed after the TA treatment. The presence of these peaks clearly points to TA, as an organic substance, being attached to the electrode surface^[^ ^12^, ^15^ ^]^ Meanwhile, as seen in Figure 1e, Raman spectra also suggest the formation of the TA‐Cu^2+^ complex based on the appearance of characteristic peaks of TA.^[^ ^16^ ^]^

X‐ray Diffraction (XRD) and X‐ray photoelectron spectroscopy (XPS) are carried out to unveil the composition of CuO NW (Figure 1f,g–i Figure S8, Supporting Information). Together, CuO and Cu_2_O in the nanowire can be found before and after TA treatment. This mixed oxidation state is beneficial for NO_3_RR.^[^ ^17^ ^]^ Meanwhile, high‐resolution XPS spectra of C1s and O1s evidence the presence of a TA layer on the surface. As shown in Figure 1g, after the TA treatment, the characteristic peak at 923.5 eV indicates the formation of the benzene ring (π–π*). Besides, the percentage of Cu─O in the O1s spectrum decreases significantly, confirming the coverage of the TA‐Cu^2+^ layer on the CuO surface.

### 2.2 NO_3_RR Performance Under Neutral/Acidic Conditions

**Figure 2:**
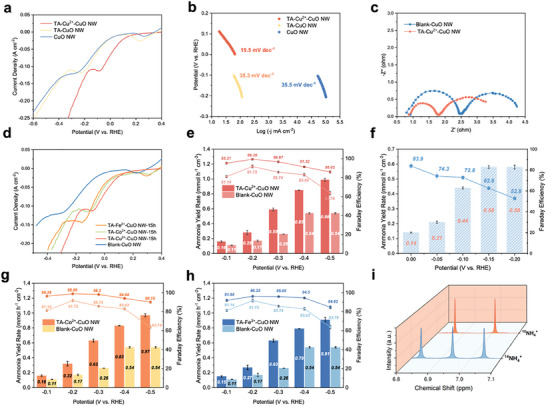
Electrocatalytic performance evaluation of NO_3_RR. a) LSV curves and b) Tafel slopes of TA‐Cu^2+^‐CuO NW/Cu foam, Blank‐CuO NW/Cu foam, and TA‐CuO NW/Cu foam. c) EIS spectra of TA‐Cu^2+^‐CuO NW/Cu foam and Blank‐CuO NW/Cu foam at −0.10 V versus RHE. d) LSV curves of TA‐M^2+^‐CuO NW/Cu foam with different metal ions (Cu^2+^, Co^2+^, Fe^2+^). e) Ammonia yield rate and FE of TA‐Cu^2+^‐CuO NW/Cu foam in neutral condition (0.5 m K_2_SO_4_ + 2000 ppm KNO_3_). f) Ammonia yield rate and FE of TA‐Cu^2+^‐CuO NW/Cu foam in acidic condition (pH = 3.0, 0.5 m K_2_SO_4_ + 2000 ppm KNO_3_ + H_2_SO_4_). g,h) Ammonia yield rate and FE of TA‐Cu^2+^‐CuO NW/Cu foam and TA‐Fe^2+^‐CuO NW/Cu foam in neutral condition. i) ^1^H NMR spectra of the electrolyte after NO_3_RR using ^15^NO_3_^−^ and ^14^NO_3_^−^.

The activity and selectivity of NO_3_RR are sensitive to the cation‐ions‐induced electric field of which a local electrostatic affection for nitrate ions is formed.^[3a]^ In this work, both the electrochemical characterizations and product analysis are employed to investigate this IEF effect on NO_3_RR. The electrochemical tests are conducted in a two‐chamber H‐cell with three‐electrode system (Figure S14, Supporting Information). The nitrate concentration contained in the electrolyte is 2000 ppm (32.3 mm) to simulate the common industrial wastewater as reported before.^[^ ^18^ ^]^ As shown in **Figure** **2**a, linear sweep voltage (LSV) curves show the enhancement of the same CuO NW/Cu foam electrode after the TA‐Cu^2+^ treatment. We also compare the samples with the TA‐Cu^2+^ layer and TA layer where with the only‐TA layer, almost no varieties could be observed suggesting that the functional group of this IEF strategy should be the cation ions fixed on the electrode surface. Meanwhile, Tafel slopes are investigated as well and suggest similar trends where the blank‐CuO NW and TA‐CuO NW have comparable values of 35.5 and 35.3 mV dec^−1^, respectively (Figure 2b). Benefiting from the IEF‐induced higher local nitrate concentration, the reaction kinetics of TA‐Cu^2+^‐CuO NW is more rapid of which the Tafel slope is decreased to 19.5 mV dec^−1^ at −0.10 V versus RHE and following the better activation kinetics as shown in EIS spectra (Figure 2c; Figure S8, Supporting Information). Moreover, the electrochemical surface area (ECSA) for the CuO NW/Cu foam electrode before and after TA treatment is investigated (Figure S10, Supporting Information). A 6‐fold increment could be observed through Electrochemical double‐layer capacity (C_dl_) with the TA‐Cu^2+^ layer (Figure S11, Supporting Information).

To elucidate the improvement of the IEF effect rather than the catalytic effect from the metal ions, LSV tests are conducted between different TA‐M^2+^ layer samples (Figure 2d). Similar electrochemical activities and ammonia yield performances (Figure 2d,e,g,h) can be observed with varying types of cations (Cu^2+^, Fe^2+^, Co^2+^). The details for ammonia detection and corresponding yield/FE calculations can be found in the Supporting information (Figure S15, Supporting Information). In the electrolyte of 0.5 m K_2_SO_4_ with 2000 KNO_3_, the gaps in ammonia yield rate and corresponding Faraday efficiency (FE) could be observed as shown in Figure 2e. At −0.2 V versus RHE, the TA‐Cu^2+^‐CuO NW reached the highest FE of 99.39%, 8% higher than blank‐CuO NW. At −0.5 V versus RHE, this gap is further enlarged to 23% while the highest ammonia yield rate has reached 0.99 mmol h^−1^ cm^−2^, a two‐fold increase compared to the blank electrode. The FE of nitrite, the key intermediate and side product, is also examined as shown in Figure S31 (Supporting Information). Additionally, in acidic conditions, the intensive content of protons in the electrolyte makes the competition for HER more severe, ultimately inhibiting the NO_3_RR. Furthermore, Cu‐based catalysts are sensitive to the acid, and suffer from the carrion issue, resulting in poor stability.^[^ ^19^ ^]^ Herein, the TA‐Cu^2+^ organo‐metallic layer is examined under an acidic electrolyte as well (pH = 3.0). At −0.1 V versus RHE, it could still maintain the FE of 72.8% superior to the pristine CuO NW/Cu foam electrode (FE of 50.3%) (Figure 2f). More encouragingly, under this condition, the TA‐Cu^2+^‐CuO NW/Cu foam worked stably for 100 h (Figure S12, Supporting Information), while the CuO NW electrode failed soon because of severe corrosion (Figure S13, Supporting Information). To further examine the acidic corrosion of the electrodes, we tested the concentration of Cu^2+^ of the electrolyte through inductively‐coupled Plasma optical emission spectrometry (ICP‐OES) after continuous 1 h operation in pH 3 electrolyte (Figure S33 and Table S4, Supporting Information). The result clearly indicates the severe Cu^2+^ dissolution of blank CuO NW/Cu foam electrode while the stability is greatly enhanced with the TA layer.

### 2.3 Mechanistic Study of IEF Effect Toward Ammonia Production Through NO_3_RR

**Figure 3:**
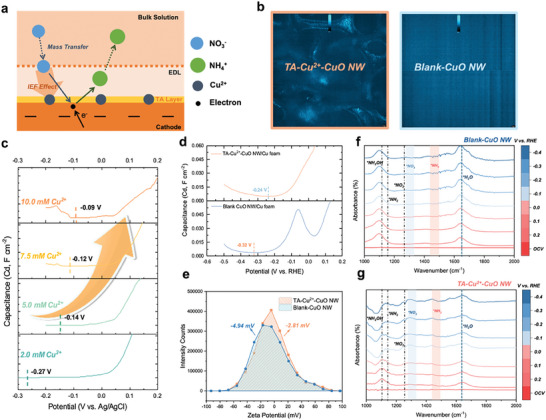
Mechanism investigation of IEF effect on TA‐Cu^2+^‐CuO NW/Cu foam. a) Schematic of proposed IEF mechanism for nitrate ions. b) Fluorescence microscopy images of TA‐Cu^2+^‐CuO NW/Cu foam and Blank‐CuO NW/Cu foam. c) Variations of PZC in electrolytes with different Cu^2+^ concentrations. d) Comparison of PZC and e) Zeta potential of TA‐Cu^2+^‐CuO NW/Cu foam and Blank‐CuO NW/Cu foam. In situ FTIR spectra at different applied potentials (OCV, 0.2 V to −0.4 V versus RHE) of f) Blank‐CuO NW/Cu foam and g) TA‐Cu^2+^‐CuO NW/Cu foam.

Herein, we proposed the IEF effect of the TA‐Cu^2+^ on the CuO NW/Cu foam cathode with the mechanism demonstrated in **Figure** **3**a. With the TA layer, cations (Cu^2+^) are anchored on the electrode surface, forming a positive‐charges‐rich environment. With these Cu^2+^ ions, the boosting NO_3_^−^ migration evokes the ions agglomeration within EDL. Meanwhile, under acidic conditions, the Cu^2+^ has a suppressing effect on competition HER where the cations could weaken the migration of H^+^ from the bulk solution to the interface (Figure S16, Supporting Information).

To clarify TA‐Cu^2+^ driving the IEF effect, the form of Cu^2+^ is investigated through a series of electrochemical experiments and spectroscopy measurements. Fluorescence microscopy is a straightforward technique to confirm the ionic existence on the electrode surface. As shown in Figure 3b, we compare the brightness images of the TA‐Cu^2+^‐CuO NW/Cu foam and Blank‐CuO NW/Cu foam using Rhodamine B Hydrazide as the fluorescent dye. With the appearance of the TA‐Cu^2+^ layer, fluorescence could be observed indicating the ionic state of Cu^2+^ at the interface. Typically, the potential of zero charges (PZC) is regarded as a direct method to determine the specific adsorption in EDL.^[^ ^20^ ^]^ With no excess charge existing on the electrode surface, PZC could reflect whether the cations are anchored at the interface, which is determined through the minimum differential capacitance curve^[3a]^ As shown in Figure 3d, the lowest points of the differential capacitance curve indicate the corresponding PZC as reported.^[^ ^21^ ^]^ In the 0.5 m K_2_SO_4_ with 2000 ppm KNO_3_ electrolyte, the PZC for TA‐Cu^2+^‐CuO NW/Cu foam and Blank‐CuO NW/Cu foam are −0.24 and −0.32 V, respectively. This positive shift of PZC suggests the presence of cations (Cu^2+^) at the interface. Specifically, with more cations at the interface, the countercharge would happen within the EDL due to the polarization of the bulk electrolyte. Based on the definition of PZC, the excess charge of the metal phase electrode should remain zero (σ_м_ = 0), thus the potential would positively shift to counterbalance the opposite charges from the diffusion layer.^[^ ^22^ ^]^ Apart from the previously reported work on specifically absorbed cations at the outer Helmholtz plane, the Cu^2+^ is anchored through the assembly process with TA to form a stabilized metal‐ion crosslinked nanostructure.^[3a,9b,12]^ Additionally, the absorption of Cu^2+^ has been simulated on the surface of Blank‐CuO NW/Cu foam by introducing CuSO_4_ to the electrolyte to validate this analysis. With the increase of Cu^2+^ concentration in the bulk electrolyte from 2.0 to 10.0 mm, a series of PZC positive shifts (from −0.27, −0.14, −0.12 to −0.09 V) could be observed in Figure 3c. These results suggest TA‐Cu^2+^ forming the electric field at the electrode‐electrolyte surface. Besides, Zeta potential is also investigated to confirm the presence of Cu^2+^ at the interface. It could be figured out from Figure 3e that with the TA‐Cu^2+^ layer on the electrode, Zeta potential increases from −4.94 to −2.81 mV, suggesting the successful adsorption of cations.^[^ ^23^ ^]^ To eliminate the effect of diffusion, the rotational disk electrode (RDE) is employed. The working electrode placed on the RDE can achieve a steady state rapidly. Herein, we conducted the RDE test of HER in 0.5 m K_2_SO_4_ and the corresponding LSV curves of TA‐Cu^2+^‐CuO NW and Blank‐CuO NW are shown in Figure S17 (Supporting Information). With the TA‐Cu^2+^ layer, the HER on the electrode would be mitigated. Besides, under the acidic condition (pH = 3.0), the competitive HER would become more severe thus suppressing the NO_3_RR. However, with this TA‐Cu^2+^ layer, the electrode clearly shows enhanced NO_3_RR performance compared to the blank samples (Figure S18, Supporting Information).

In situ Fourier transform infrared (FTIR) spectroscopy was applied to investigate the mechanism insights of the proposed IEF effect, which presents the direct information of the absorbed species at the electrode‐electrolyte interface. Figure 3f,g depicts the FTIR absorbance spectra of TA‐Cu^2+^‐CuO NW and Blank‐CuO NW at a series of different potentials from open circuit voltage (OCV), and 0.2 to −0.4 V versus RHE, respectively. The stretching vibration of the chemical bonds could strongly suggest the presence of the specific absorbed molecules and it has been reported that the upward bands are usually ascribed to the consumption of reactants/intermediates.^[^ ^24^ ^]^ Compare the electrodes with/without the TA‐Cu^2+^ layer on the surface, the upward bands at 1320 cm^−1^ referred to the N–O asymmetric stretching appear when applying the TA‐Cu^2+^‐CuO NW electrode, which represents the better absorption of nitrate ions (*NO_3_) and furtherly implies the local nitrate accumulation. In contrast, no apparent band related to *NO_3_ is detected on Blank‐TA‐CuO NW. Meanwhile, the intensity of this *NO_3_ band increases with the applied potentials, suggesting the continuous consumption of nitrate ions during the NO_3_RR process.^[^ ^25^ ^]^ The downward bands related to the produced ammonia (*NH_3_) at 1468 cm^−1^ are more intensified on the TA‐Cu^2+^‐CuO NW electrode after normalization. A series of N‐related intermediates (*NH_2_ ≈ 1155 cm^−1^, *NH_2_OH ≈ 1115 cm^−1^, and *NO_2_ ≈ 1236 cm^−1^) could be observed as well.^[^ ^26^ ^]^ It should be noted that the shift from a downward to an upward trend in the band associated with the *NH_2_ indicates that, with the application of higher potentials, the *NH_2_ intermediate is consumed more rapidly on the TA‐Cu^2+^‐CuO NW sample. This observation strongly suggests an enhancement in the kinetics of NO_3_RR toward NH_3_. In addition, the upward bands at 1635 cm^−1^ related to the vibration of O–H could be assigned to the absorption of the H_2_O molecule. For the blank sample, the intensity increased with more negative potential, indicating the competence of HER particularly at higher reaction rates. Notably, the TA‐Cu^2+^ layer mitigates this tendency, suggesting a suppression of the competitive HER, in alignment with observations from our previous experiments.

**Figure 4:**
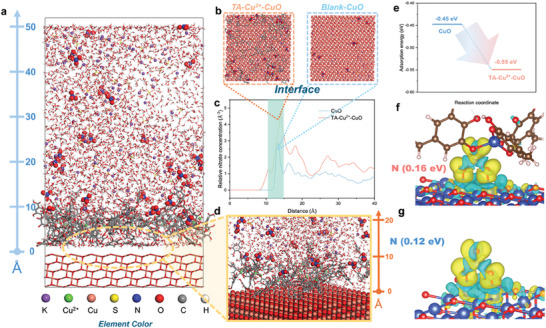
Computational simulations of IEF effects on TA‐Cu^2+^‐CuO. a) MD simulation model of the CuO (111) plane with TA‐Cu^2+^ in 0.5 m K_2_SO_4_ + 2000 ppm KNO_3_ solution. b) Interface images and c) Number of nitrate ions along the z‐axis from the electrode surface of TA‐Cu^2+^‐CuO and Blank‐CuO surfaces based on MD simulations. d) Zoomed‐in view of the electrode‐solution interface. e) Comparison of adsorption energies of *NO_3_. Differential charge density distribution of *NO_3_ on f) TA‐Cu^2+^‐CuO and g) Blank‐CuO surfaces based on DFT simulation.

The molecular dynamics (MD) simulation is performed to quantify the dynamic behavior of nitrate ions at the electrode‐electrolyte interface within the range of 0–50 Å. The comparison is made between the CuO (111) plane with or without TA‐Cu^2+^ complex in the environment of 0.5 m K_2_SO_4_ with 2000 ppm KNO_3_ to simulate the experimental environment as possible. The model of CuO (111) plane and simplified model of TA‐Cu^2+^ structure is presented in Figures S34 and S35 (Supporting Information). With the appearance of the TA‐Cu^2+^ complex layer, MD gives a straightforward presentation of nitrate ions accumulation in the surface region, which could be observed through the full model and partially enlarged snapshot (**Figure** **4**a,d). In contrast, in the absence of the TA‐Cu^2+^ complex on the CuO (111) plane, this phenomenon of local accumulation could not be observed where the ion distribution is relatively homogeneous (Figure S19, Supporting Information). The top views for the interfaces are shown in Figure 4b, which clearly indicates the “smart chosen” of nitrate ions at the TA‐Cu^2+^ interface. The relative concentration of NO_3_^−^ is investigated quantitively. At the distance of 10–15Å from the electrode surface, the maximum NO_3_^−^ number reached 5 Å^−3^ for the TA‐Cu^2+^‐CuO surface, while this number is only half for the Blank‐CuO plane (Figure 4c). Meanwhile, the number of K^+^ relative density is decreased in the range of 10–15Å at the interface with the TA‐Cu^2+^ layer (Figure S20, Supporting Information). This phenomenon arises from the neutralization of NO_3_^−^ ions by negative charges at the electrode‐electrolyte interface, as evidenced by the modulation effect of IEF on local ion concentration. In addition, to directly elucidate the IEF effect and to exclude the possible interference of alkali cations, we further performed several complementary experiments using 10 mm HNO_3_ as the electrolyte. The results showed that the TA‐Cu^2+^ layer significantly improved NO_3_RR performance and ammonia production, supporting the IEF effect (Figure S32, Supporting Information).

Combining the previous experiments and MD simulation results, we could conclude that the TA‐Cu^2+^ complex successfully forms an interfacial electric field at the interface which enables the local high concentration of nitrate ions, thus promoting the kinetics for low‐concentrated NO_3_RR. Furthermore, density functional theory (DFT) calculations are conducted to examine the thermodynamic properties of TA‐Cu^2+^‐CuO NW/Cu foam during NO_3_RR. As shown in Figure 4e, with the TA‐Cu^2+^ layer, the adsorption energy for NO_3_^−^ is reduced from −0.45 to −0.55 eV, indicating the lower energy barrier for the adsorption process from NO_3_^−^ to *NO_3_. Besides, the bonding configuration of nitrate is presented by differential charge density distribution (Figure 4f,g). With the calculation of the Bader charge, a slight enhancement could be observed in the bonding capacity. Specifically, 0.16 e^−^ from Cu d orbital is transferred to N 2p orbital with TA‐Cu^2+^ layer, which is 0.04 e^−^ higher compared to the blank electrode.

Overall, the MD simulation verified the NO_3_^−^ accumulation at the interface leading to the enhanced NO_3_RR kinetics, while DFT calculations further examined the promotion in thermodynamics aspects. It is surprising to find out that the TA‐Cu^2+^ has a positive effect on the absorbance process from NO_3_^−^ to *NO_3_, which also confirms the benefits of interfacial nitrate accumulation in NO_3_RR. All these enhancements contribute to the mechanism explanation for our proposed IEF effect.

### 2.4 Practical Application of Ammonia Production in the Flow‐Cell Reactor

**Figure 5:**
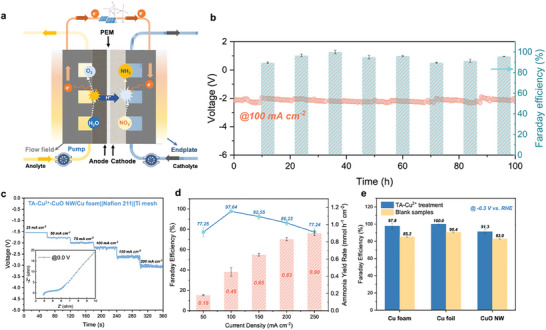
Practical ammonia production using TA‐Cu^2+^ treated electrodes in the flow‐cell reactor. a) Schematic of the working principle. b) Long‐term stability of TA‐Cu^2+^‐CuO NW/Cu foam at 100 mA cm^−2^. c) Voltage–time curve at step current densities (inset: EIS spectrum for the flow‐cell reactor). d) Ammonia yield rate and corresponding FE in neutral conditions. e) Ammonia FE of TA‐Cu^2+^ treated commercial electrode materials.

The advantage of this smart IEF strategy for electrode modulation in NO_3_RR enables the further exploration of its flow‐cell application toward reality. It has been reported that alkaline NO_3_RR shows great application potential in ammonia production due to its relatively mild HER. Nevertheless, in neutral and acidic environments, NO_3_RR may encounter challenges in achieving practical ammonia yield due to the increased competition from HER, especially when faced with the condition of low nitrate concentration from wastewater. With this IEF strategy, the nitrate ions are smartly chosen by the TA‐M^2+^ layer thus solving the obstacle originating from the competitive HER and low nitrate concentration. Moreover, we promote this strategy to the direct surface modulation of commercial Cu foam and Cu foil electrodes (Figures S21–S26, Supporting Information). Under the same condition, all the samples show an enhancement of over 10% in nitrate‐to‐ammonia FE (**Figure** **5**e).

On such promising ground, we look ahead to the use of this strategy for electrode surface treatment and further application in the flow‐cell reactor for ammonia production (Figure S27, Supporting Information). Compared with a static H‐cell reactor, the flow‐cell reactor could enhance the mass transfer for the reactants and products, providing stable conditions for the reaction. Specifically, surface‐modulated TA‐Cu^2+^‐CuO NW/Cu foam is utilized as the cathode electrode and commercial Ti mesh is chosen as the anode. A proton exchange membrane (Nafion 211) is utilized to separate the catholyte and anolyte, preventing the oxidation of NH_3_ on the anode. In this flow system, the electrolytes are fed into the flow cell through two peristaltic pumps. The supportive electrolyte for both the cathode and anode is 0.5 m K_2_SO_4_ to improve the conductivity. Meanwhile, 2000 ppm KNO_3_ is added to the catholyte as the reactant for NO_3_RR. The schematic illustration of the working principle for the flow‐cell reactor is depicted in Figure 5a. The stability of the whole system as well as the cathode electrode itself is examined through the long‐term operation test. For the flow‐cell reactor, systematic stability is the prerequisite for the practical application and is determined by the stability of the individual components, such as catalyst, working conditions, cell components, etc.^[^ ^27^ ^]^ As shown in Figure 5b, the flow‐cell reactor maintains stability of over 100 h along with a stable FE of over 90% for ammonia production under neutral conditions. After the 100 h operation test, the TA‐Cu^2+^‐CuO NW/Cu foam is examined through XPS, FTIR and Raman Spectroscopy, indicating good chemical stability of the electrode (Figures S28–S30, Supporting Information). Meanwhile, the electrochemical performance and ammonia production performance at various current densities (from 50 to 250 mA cm^−2^) are investigated as well (Figure 5c,d). The highest NO_3_RR FE for ammonia production could reach 97.64% at 100 mA cm^−2^ and maintain 86.22% at 200 mA cm^−2^, while the highest ammonia yield rate appears at 250 mA cm^−2^, with a peak of 0.9 mmol h^−1^ cm^−2^. As a result, with this smart IEF strategy, we enable the common electrodes to reach great NO_3_RR performance under low nitrate concentration and neutral conditions, which is even comparable to the alkaline NO_3_RR.

## 3 Conclusion

In our work, we propose a smart IEF electrode modulation strategy through TA‐M^2+^ coordinated nanostructure. With the TA‐M^2+^ layer on the electrode surface, the forming relatively positively charged electric field is demonstrated thus inducing the increase in local nitrate concentration at the electrode‐electrolyte interface. This strategy greatly benefits the kinetics and enhances the selectivity toward low‐concentrated NO_3_RR, which is proven with the highest FE of 99.38% at −0.2 V versus RHE and the highest ammonia yield rate of 0.99 mmol h^−1^ cm^−2^ at −0.5 V versus RHE under neutral condition (TA‐Cu^2+^‐CuO NW/Cu foam). Compared to the blank electrode without this TA‐M^2+^ coordinated nanostructure, a two‐fold increase in ammonia yield rate could be observed and this gap enlarged under more negative potentials. Notably, even in the acidic electrolyte of pH 3.0, the ammonia FE could maintain 83.9% at 0.0 V versus RHE with a yield rate of 0.14 mmol h^−1^ cm^−2^. Such an IEF effect is verified through both experimental and computational methods. Furthermore, the practical application of commercial materials and the flow‐cell reactor are investigated. In summary, this work is anticipated to inspire fresh insights into the impact of the IEF on NO_3_RR and potentially extend to other electrochemical processes, particularly those involving various intermediates and by‐products.

## Conflict of Interest

The authors declare no conflicts of interest.

## Author Contributions

L.A. and X.Z. proposed the topic. K.Z. designed and conducted the experiment and drafted the manuscript. Y.X. conducted the theoretical calculation. All authors contributed to the editing of the manuscript.

## Supporting information



Supporting Information

Supplemental Movie 1

## Data Availability

The data that support the findings of this study are available from the corresponding author upon reasonable request.
