# Supplementary material for: Leveraging Interfacial Electric Field for Smart Modulation of Electrode Surface in Nitrate to Ammonia Conversion
Source: Adv Sci (Weinh). 2024 Dec 2;12(4):2410763. doi: 10.1002/advs.202410763 (PMC11775551; doi:10.1002/advs.202410763)
Supplement: Supplementary file 1 — Supporting Information [file ADVS-12-2410763-s001.docx]

**Leveraging Interfacial Electric Field for Smart Modulation of Electrode Surface in Nitrate to Ammonia Conversion**

**Supplementary information**

Kouer Zhang^a^, Yifan Xu^b^, Fatang Liu^a, c^, Qing Wang^a^, Xiaohong Zou^a^, Mingcong Tang^a^, Michael K.H. Leung^b, d^, Zhimin Ao^e^, Xunhua Zhao^f^, Xiao Zhang^a, g, *^ and Liang An^a, g, *^

*^a^ Department of Mechanical Engineering, The Hong Kong Polytechnic University, Hung Hom, Kowloon, Hong Kong SAR, China*

*^b^ Ability R&D Energy Research Centre, School of Energy and Environment, City University of Hong Kong, Kowloon, Hong Kong SAR, China*

*^c^ College of Chemistry and Chemical Engineering, Northeast Petroleum University, Daqing 163318, China*

*^d^ State Key Laboratory of Marine Pollution, City University of Hong Kong, Hong Kong SAR, China*

*^e^ Advanced Interdisciplinary Institute of Environment and Ecology, Guangdong Provincial Key Laboratory of Wastewater Information Analysis and Early Warning, Beijing Normal University, Zhuhai 519087, China*

*^f^ Key Laboratory of Quantum Materials and Devices of Ministry of Education School of Physics, Southeast University, Nanjing 211189, China*

*^g^ Research Institute for Smart Energy, The Hong Kong Polytechnic University, Hung Hom, Kowloon, Hong Kong SAR, China*

**Experimental section:**

**Reactants:**

Anhydrous ethanol (CH_3_CH_2_OH), hydrochloric acid (HCl), and sulfuric acid (H_2_SO_4_) were all purchased from Anaqua (ACS reagent). Sodium hydroxide (NaOH), ammonium persulphate ((NH_4_)_2_S_2_O_8_), potassium sulfate (K_2_SO_4_), and potassium nitrate (KNO_3_) were purchased from Sigma-Aldrich (ACS reagent, ≥99.0%). Cupric sulfate pentahydrate (Cu_2_SO_4_·5H_2_O)，cobalt(II) sulfate heptahydrate (CoSO_4_·7H_2_O), Iron sulfate heptahydrate (FeSO_4_·7H_2_O) were purchased from Macklin (ACS reagent, ≥99.0%). Tannic acid (C_76_H_52_O_46_, ACS reagent) was purchased from Thermo Scientific (Alfa Aesar). Salicylic acid and sodium citrate were purchased from Dieckmann (ACS reagent, ≥99.0%). Sodium hypochlorite (NaClO) and sodium nitroferricyanide (C_5_FeN_6_Na_2_O) were purchased from Macklin (ACS reagent, ≥99.0%). Dimethyl sulfoxide-d6 (DMSO-d6) was purchased from Sigma-Aldrich (99.9 atom %D). All reactants were used without any further purification. All aqueous solutions were prepared with deionized water (resistivity over 18 MΩ cm at 25 °C, Millipore).

**Electrode fabrication:**

Firstly, the commercial Cu foam was first cut into the size of 1 cm $\text{×}$ 2 cm, and then washed with anhydrous ethanol and 1.0 M hydrochloric acid through ultrasonic method. Secondly, the Cu foam was soaked in 0.1 M ammonium persulfate and 1.0 M sodium hydroxide solution for 1 hour at room temperature. This method enabled in situ etching to grow uniform Cu(OH)_2_ nanowires (NWs) directly from the Cu foam. Then, the samples were annealed in a flowing Ar atmosphere with the flow rate of 100 sccm at 200 °C for 2 hours to synthesized CuO NW/Cu foam.

**Tannic acid electrode modification:**

The fabricated CuO NW/Cu foam electrode then went through the modification process of tannic acid and metal ions (TA-M^2+^). The TA-M^2+^ layer was synthesized by the coordination of metal ions (Cu^2+^, Fe^2+^ and Co^2+^) with TA. Following the typical procedure for TA coordination, 0.108 g TA and 0.01 M metal-sulfate were dissolved into 200 mL deionized water and stirred to form the homogenous solution. After that, 1.0 M KOH was slowly dropped into the solution until the pH was adjusted to 8.5. The CuO NW/Cu foam electrode was placed in the above solution for 15 h and then washed with anhydrous ethanol and deionized water thoroughly.

**Material characterizations:**

X-Ray diffraction analysis (XRD) data were collected on a Rigaku Smart Lab X-ray diffractometer with Cu Kα radiation (Rigaku SmartLab 9kW). Scanning electron microscopy (SEM) was performed on TESCAN MIRA, which is a high-resolution (HR) analytical SEM with a high brightness field emission electron source (FEG). Energy-dispersive X-ray spectroscopy (EDS) elemental mapping of SEM was performed on Tescan VEGA3 with energy dispersive X-ray spectroscopy (EDX) detector. Transmission electron microscopy (TEM), high-resolution TEM, HAADF-STEM, high-resolution HAADF-STEM and EDS elemental mapping were performed using a multipurpose JEOL JEM-2100F with field emission of 200 Kev. X-ray photoelectron spectroscopy (XPS) data were measured on Thermo Fisher Scientific Nexsa using monochromatic Al Kα radiation (1,486.6 eV). The XPS spectra were calibrated based on the detected carbon C 1s peak to 284.6 eV. The Raman spectra were performed on Renishaw Micro-Raman spectroscopy system and Fourier transform infrared (FTIR) spectra were conducted through Bruker Vertex-70 FTIR spectroscopy. The in-situ FTIR spectroscopy tests were conducted correlatedly with CHI760e. Fluorescence microscope was conducted through Olympus BX51 using the Rhodamine B hydrazide as fluorescence probe.

**Electrochemical characterizations:**

The electrochemical performance tests were first carried out in a H-cell using Nafion 211 as the ion-exchange membrane. In this three-electrode system, the samples synthesized in this work (1.0 cm $\text{×}$ 1.0 cm) were chosen as the working electrode (cathode), while the Pt foil (1.0 cm $\text{×}$ 1.0 cm) was chosen as the counter electrode (anode) and Ag/AgCl electrode (with 1.0 M KCl solution) was chosen as the reference electrode. The catholyte was 0.5 M K_2_SO_4_ and 2000 ppm KNO_3_ for the cathode, while the anolyte remained 0.5 M K_2_SO_4_ if not specified. The electrochemical performance data for the three-electrode system were collected and analyzed through CHI 760e workstation. All the potentials measured against the Ag/AgCl electrode in this work were converted to the reversible hydrogen electrode ($\text{RHE}$) scale through the equation below:

$$\text{E}\text{ }\left( \text{vs. RHE} \right)\text{ = E }\left( \text{vs. Hg/HgO} \right)\text{ + 0.2224 V + 0.0591 × pH}$$

The pH value of 0.5 M K_2_SO_4_ solution used in this work is 7.0 determined by pH meter (LICHEN pH-100A). The linear sweep voltammetry (LSV) was adopted with 90% IR-compensation of potential. The inner resistance was measured by the potentiostatic electrochemical impedance spectroscopy (EIS) at frequencies from 0.1 Hz to 100 kHz. The electrochemical active surface area ($\text{ECSA}$) was determined through the estimation of electrochemical double-layer capacitance (C_dl_) of the catalytic surface. The $\text{ECSA}$ was calculated according to the following equations:

$\text{ECSA = }\frac{\text{C}_{\text{dl}}}{\text{C}_{\text{s}}}$

$\text{C}_{\text{dl}}\text{ = }\frac{\text{i}_{\text{c}}}{\text{v}}$

In the above equations, C_s_ represented the specific capacitance (C_s_ = 0.040 mF cm^-2^); i_c_ is the value of the charging current density (mA cm^-2^) which is the half of the charge/discharge current difference; v is the scan rate (mV s^-1^). The cyclic voltammetry (CV) curves in C_dl_ determination were measured in a potential window without Faradaic current occurred in the electrolyte of 1.0 M KOH under incremental scanning rates of 20 mV s^-1^, 40 mV s^-1^, 60 mV s^-1^, 80 mV s^-1^, and 100 mV s^-1^, respectively.

**Product detection:**

The concentration of ammonia is decided through the technique of ultraviolet (UV) absorption spectroscopy. Indophenol blue spectrophotometric method was chosen in this work for ammonia detection. Three color reagents were prepared in advance, which were named as solution A, solution B and solution C. Solution A is a mixture of 1.0 M NaOH, 5 wt% salicylic acid and 5 wt% sodium citrate solution. Solution B is 0.05 M Sodium hypochlorite (NaClO) and solution C is 0.05 M sodium nitroferricyanide (C_5_FeN_6_Na_2_O). Before the test in UV, the sample electrolyte was diluted to the detection range. After that, 1.0 ml of dilution electrolyte was taken and 1.0 ml solution A, 0.5 ml solution B, 0.1 ml solution C were added sequentially and placed in a light-proof environment for 2 hours. Finally, the solution was measured in UV-vis spectrophotometry (Dynamica Halo-DB20). To be mentioned, the standard concentration-absorbance curve was calibrated using different concentration of standard NH_4_Cl solution. The peak for the formation of indophenol blue appeared at the wavelength of 655 nm.

In addition, isotope-labelling measurement was also adopted to prove the source of ammonia produced in this work. The ^1^H NMR (500MHz SB Liquid Bruker Advance NMR Spectrometer) method was chosen using DMSO-d6 as solvent.

The concentration of nitrate was detected in this work through the technique of ultraviolet (UV) absorption spectroscopy. Firstly, samples were collected and diluted to the detection range. Next, 40.0 µl of 1.0 M hydrochloric acid and 4.0 µl of 0.8wt% sulfamic acid solution were added to 2.0ml of diluted sample. After placing for 30 minutes, the absorption intensities at wavelengths of 220 and 275 nm were recorded. The absorbance was calculated according to the formula below:

$$\text{A = A}_{\text{220nm}}\text{ – }\text{A}_{\text{275nm}}$$

The concentration-absorbance curve was calibrated using a series of standard potassium nitrate solutions which were linearly fitted. The concentration of nitrate was then calculated from the nitrate concentration-absorbance curve.

**Ammonia Faraday efficiency (FE), energy efficiency (EE) and yield rate calculations:**

The FE for ammonia production was calculated based on the following equations:

$\text{FE = }\frac{\text{n × c × V × F}}{\text{Q}}$ (H-cell reactor)

$\text{FE = }\frac{\text{n × c × v × F}}{\text{I}}$ (Flow-cell reactor)

The yield rate of ammonia is calculated with the equation below:

$\text{Y}_{\text{N}\text{H}_{\text{3}}}\text{ =} \frac{\text{c}}{\text{t}}$ (H-cell reactor)

$\text{Y}_{\text{N}\text{H}_{\text{3}}}\text{ = c × ν}$ (Flow-cell reactor)

In the above equations, n is the electron transfer number of the reaction to form ammonia (n = 8 for NO_3_RR); cis the concentration of the product (mol mL^-1^); v is the flow rate of the cathode electrolyte (mol mL^-1^ s^-1^); t is the reaction time (h); F is the Faraday constant (F = 96485 C mol^-1^); Vis the volume of cathode electrolyte in H-cell reactor (mL); Q is the total consumed amount of charge (C); I is the current of flow-cell reactor (A).

**Determination of potential of zero charge (PZC):**

The PZC of Blank-CuO NW/Cu foam electrode and TA-Cu^2+^-CuO NW/Cu foam was tested through minimum differential capacitance measurements in the three-electrode system consisting of a Pt foil as the cathode, an Ag/AgCl electrode as the reference electrode, and 0.5 M K_2_SO_4_ with 2000 ppm KNO_3_ as the electrolyte. The electrochemical impedance-potential spectra were measured at a frequency of 1 Hz with a sinusoidal voltage perturbation of 5 mV and a potential increment of 5 mV by the electrochemical workstation (CHI 760e). Besides, to verify the effect Cu^2+^ on the PZC, the PZC of the CuO NW/Cu foam was determined by the minimum differential capacitance in a series of CuSO_4_ solutions containing 2 mM, 5 mM, 7.5 mM, 10 mM under Ar-statured condition. The PZC values were determined from the minimum value of the specific capacitance (C_d_), calculated from the imaginary part (Z”) of the impedance spectra and angular frequency (ω, 1 Hz) according to the following equation:

$$\text{C}_{\text{d}}\text{ = |1/}\text{ωZ}\text{''|}$$

**Molecular dynamic simulation:**

Molecular dynamic (MD) simulations were applied to investigate the concentration distribution of K^+^ and NO_3_^-^ on CuO (1 1 1) surface with or not complex Cu^2+^-TA insertion. The solid-liquid interface calculations were conducted with COMPASS Ⅲ force field using the Forcite module in Materials Studio (MS) 2020(1, 2). The dimensional lengths of the CuO (1 1 1) slab are 46.15 Å × 47.96 Å in xoy plane (Figure S28). The solution contains 30 KNO_3_, 80 K_2_SO_4_ and 3704 H_2_O molecules in a rectangular box with length scales of 46.15 Å × 47.96 Å × 51.97 Å. After geometry optimization, the solutions were respectively placed on the optimized pristine and Cu^2+^-TA complex covered CuO (1 1 1) surface (Figure S29). All MD calculations were performed under the NVT ensemble (T=298.0 K) with a time step of 1 fs and a total simulation time of 500 ps, during which simulation trajectories were recorded every 5000 steps. The running time was long enough for system energy and temperature reaching stable. The temperature was controlling by a Nose-Hoover thermostat. The Ewald scheme and atom-based cutoff method (*i.e.*, a radius of 12.5 Å) were applied to treat electrostatic and van der Waals (vdW) interactions, respectively. The concentration profiles of K^+^ and NO_3_^-^ along perpendicular direction were analyzed. The solid-liquid interfaces snapshots were also displayed.

**Density functional theory calculations:**

Our spin-polarized density functional theory (DFT) calculations were conducted using the Vienna Ab Initio Package (VASP), employing the generalized gradient approximation (GGA) with the Perdew-Burke-Ernzerhof (PBE) functional(3-5). A cutoff energy of 400 eV was applied for plane wave descriptions, and the Brillouin zone was sampled with a 1×1×1 Γ-centered Monkhorst-Pack k-point mesh in all calculations. To model the Cu(111) surfaces, we used a slab model with four atomic layers and a vacuum thickness exceeding 15 Å, arranged in a 3 × 3 × 1 supercell. All adsorbed species were optimized with the convergence of energy and force less than 10^-5^ eV and 0.01 eV/Å, respectively. Van der Waals interactions were treated using the DFT-D3 method with BJ-Damping(6).

The free energy changes were calculated using the following equation(7, 8):

ΔG = ΔE + ΔZPE – TΔS

Here, ∆E represents the adsorption energy, and ∆ZPE signifies the change in zero-point energy. ∆S represents the entropy change for each reaction, with T held constant at 298.15 K in this study. In DFT calculations, it can be challenging to intuitively represent the energy of electronegative ions like OH^-^ and NO_3_^-^. Therefore, the energy of charged OH^-^ is transformed using the following formula: ΔG(OH^-^) = ΔG(H_2_O) − ΔG(1/2 H_2_). On the other hand, the free energy of NO_3_^-^ can be determined using the following equations(9, 10):

NO_3_^-^_(l)_ + H^+^ → HNO_3(l)_

HNO_3(l)_ → HNO_3(g)_

According to the CRC Handbook of Chemistry and Physics(11), ΔG_1_ = 0.317 eV and ΔG_2_ = 0.074 eV. Consequently, the free energy change for NO_3_^-^ can be calculated using the following equation:

ΔG(NO_3_^-^) = ΔG(HNO_3(g)_) − 1/2ΔG(H_2_) − 0.392 eV

**Supplementary figures:**


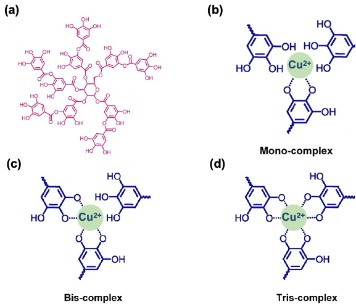


**Figure S1.** The state of TA-Cu^2+^ structures under different pH conditions. a) The molecular structure of TA b) mono-complex (pH < 2), c) bis-complex (3< pH < 6) and d) tris-complex (pH > 7) structures.

**Figure S2.** LSV curves of time-related TA-Cu^2+^-CuO NW/Cu foam samples.


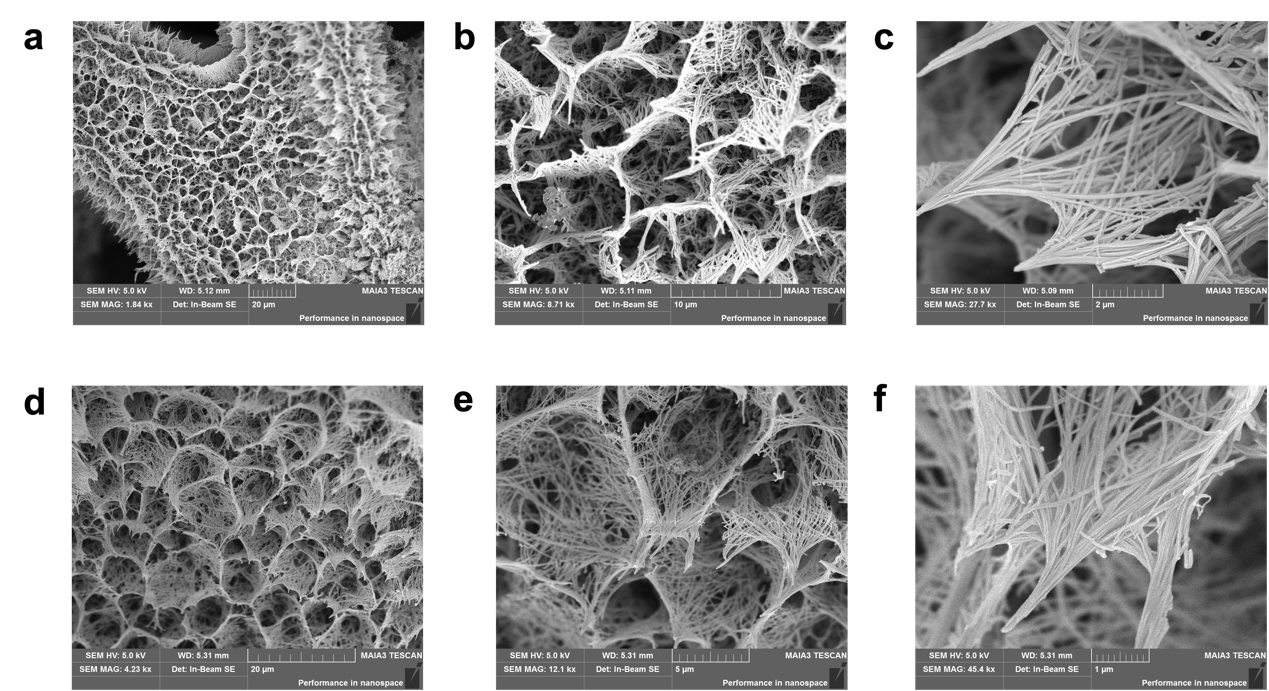


**Figure S3.** SEM images of a, b, c) TA-Cu^2+^-CuO NW/Cu foam and d, e, f) blank-CuO NW/Cu foam.


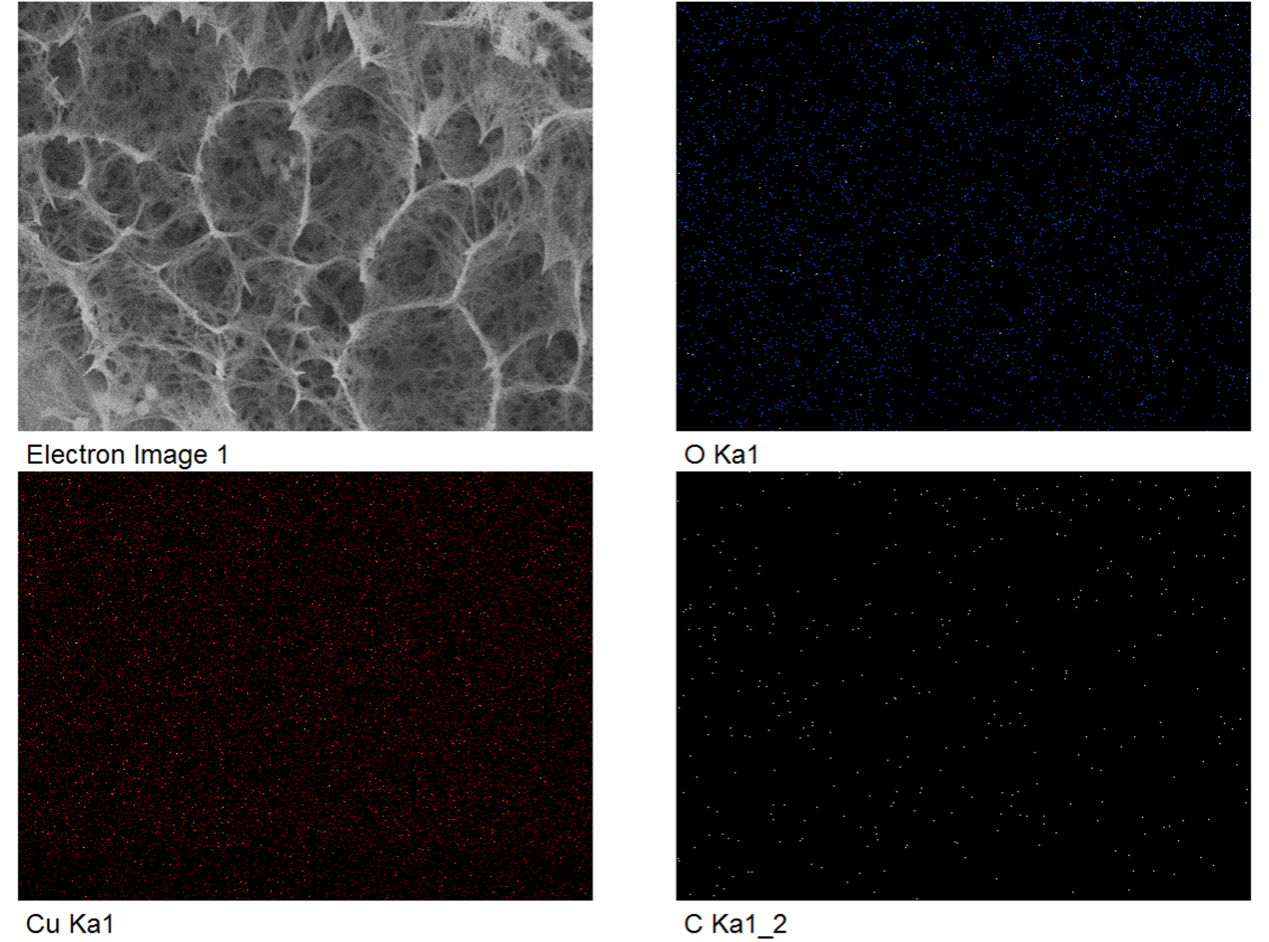


**Figure S4.** SEM mappings of TA-Cu^2+^-CuO NW/Cu foam.


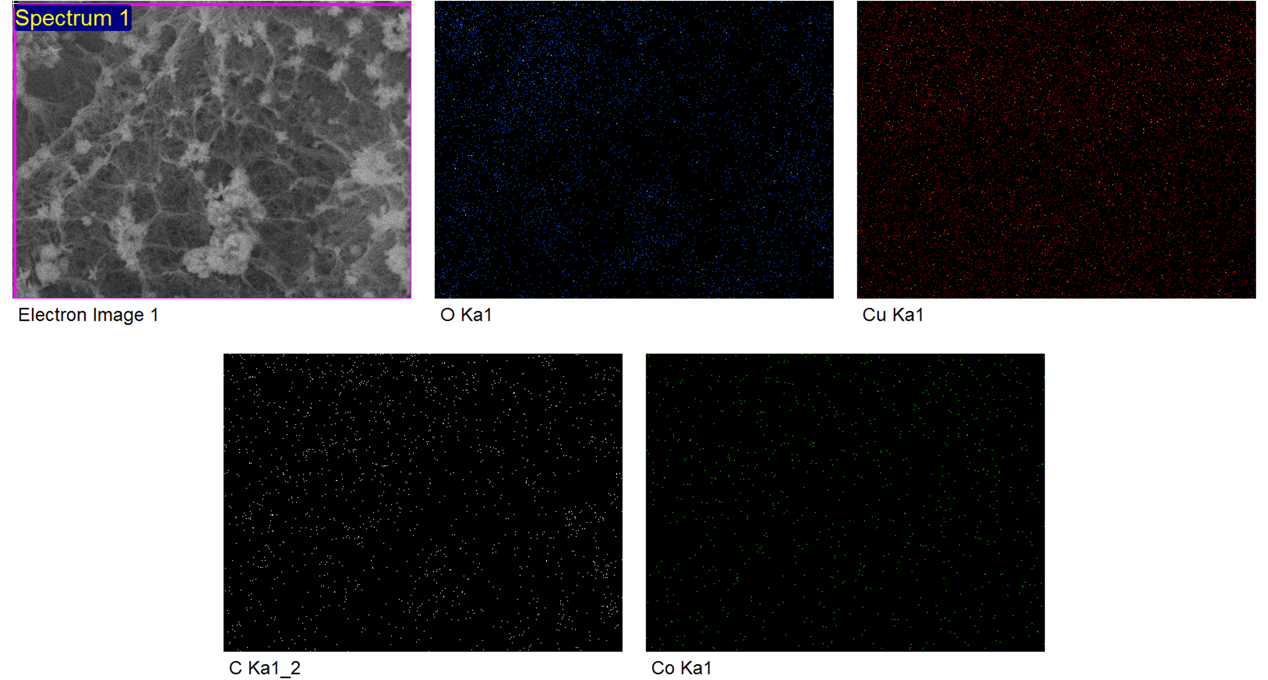

**Figure S5.** SEM mappings of TA-Co^2+^-CuO NW/Cu foam to prove the existence of added metal ions on the electrode surface.

**
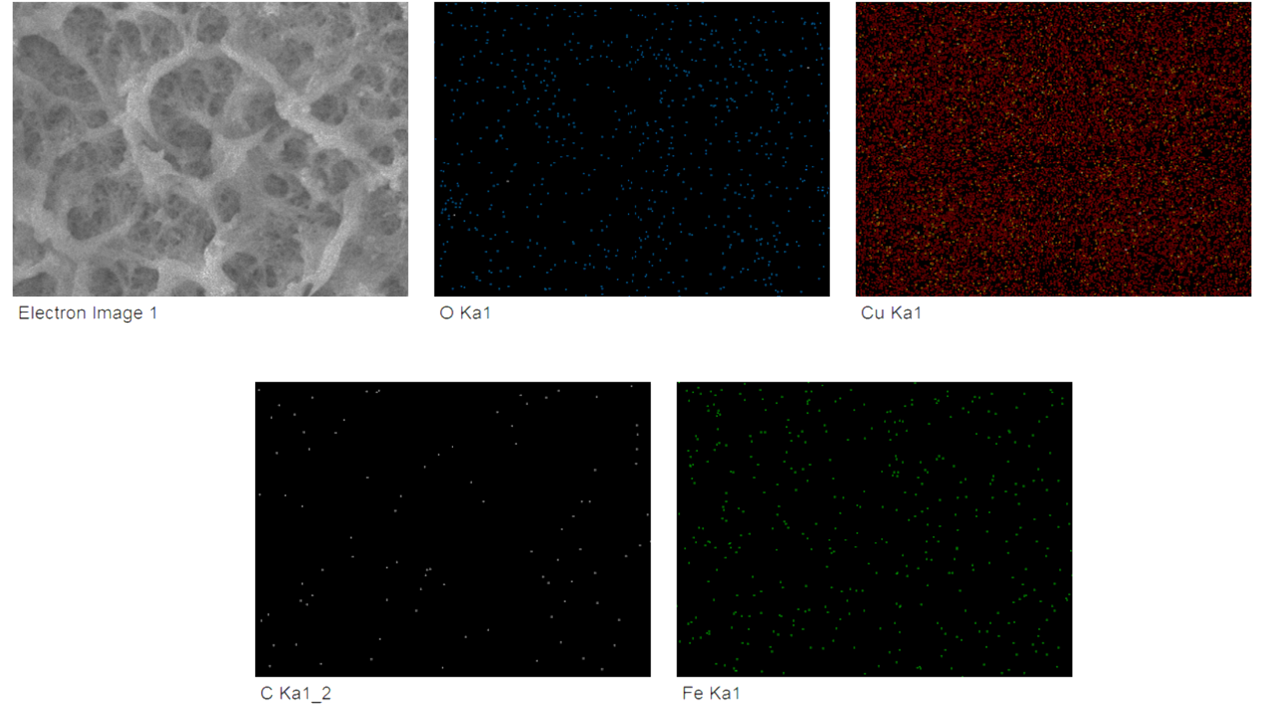
**

**Figure S6.** SEM mappings of TA-Fe^2+^-CuO NW/Cu foam to prove the existence of added metal ions on the electrode surface.


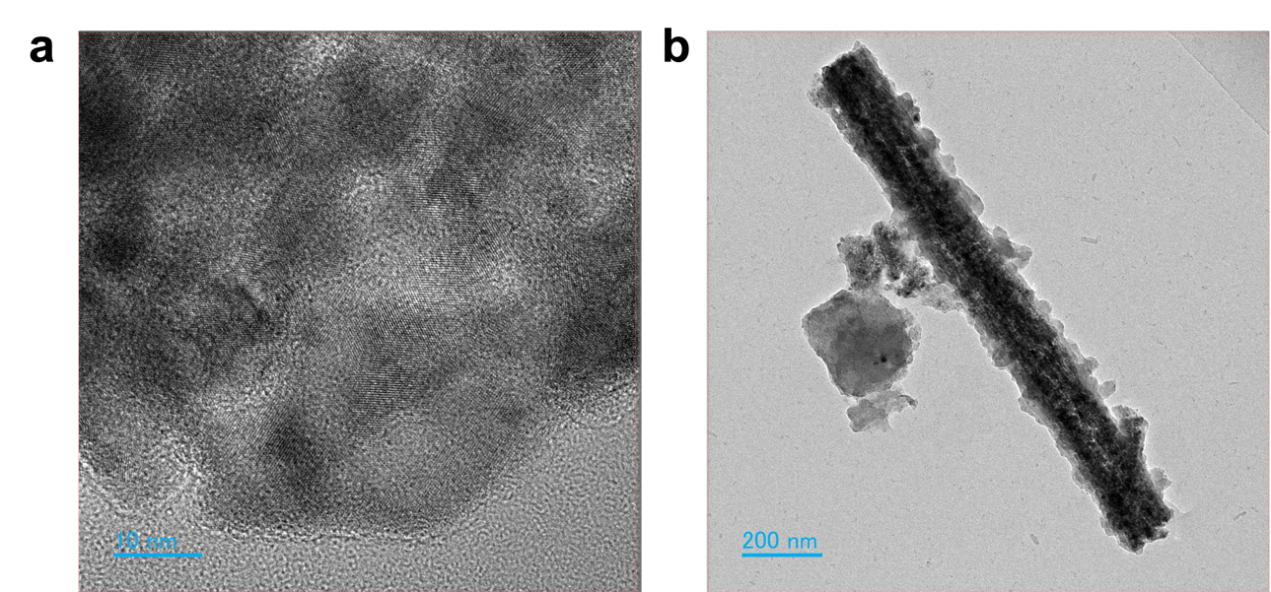


**Figure S7.** a) HRTEM image of TA-Cu^2+^-CuO NW and b) Bright-field TEM images of TA-Cu^2+^-CuO NW .


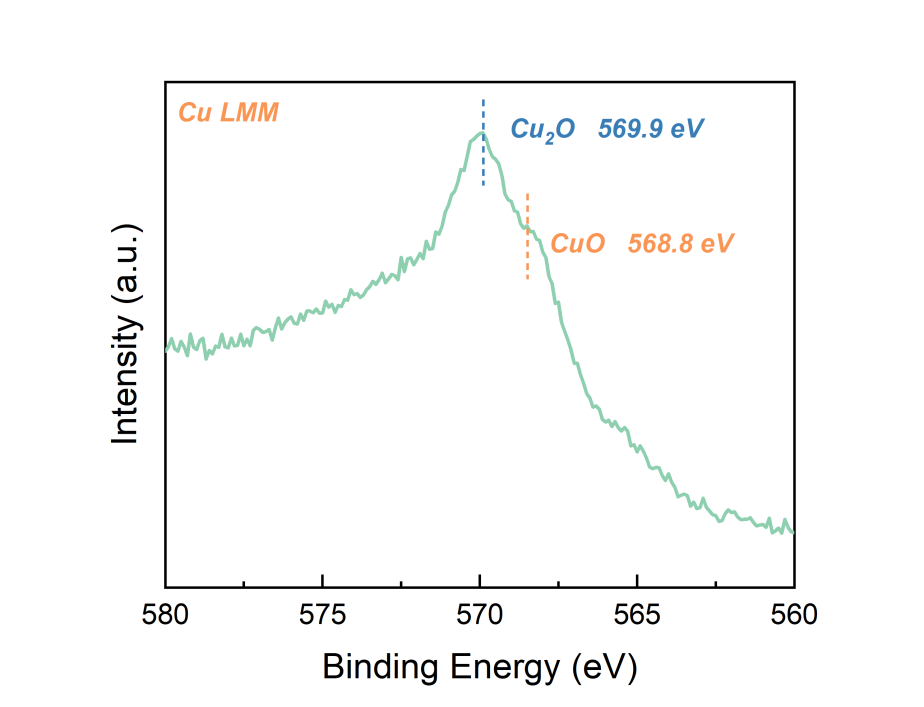


**Figure S8.** XPS spectra of Cu LMM of TA-Cu^2+^-CuO NW.


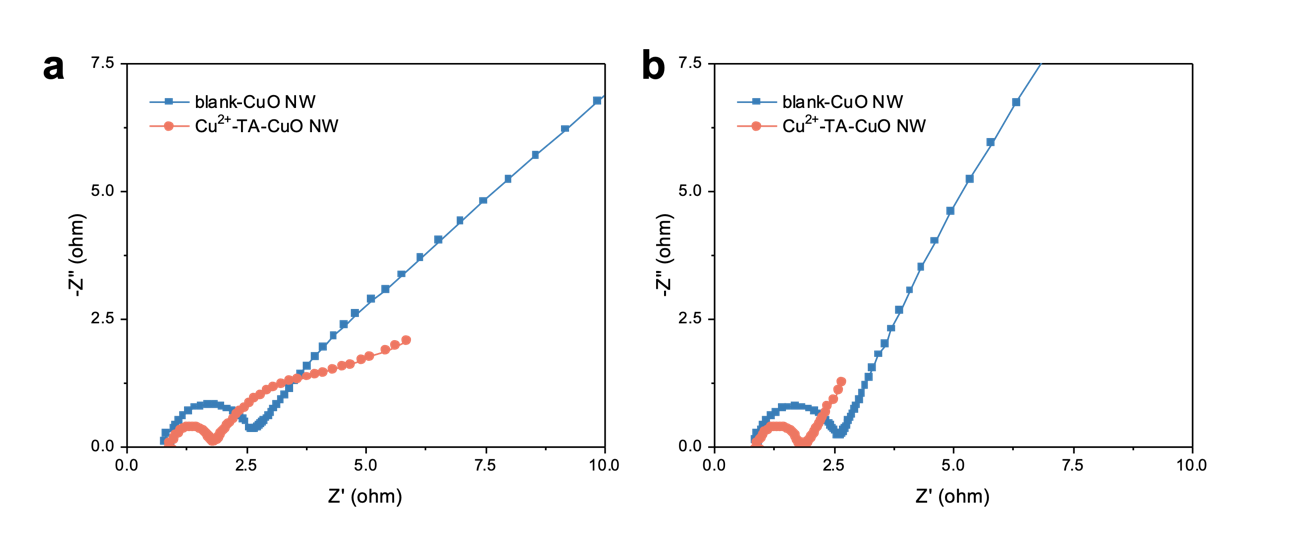


**Figure S9.** EIS spectra of TA-Cu^2+^-CuO NW/Cu foam and Blank-CuO NW/Cu foam at a) -0.05 V vs. RHE and b) 0.00 V vs. RHE.


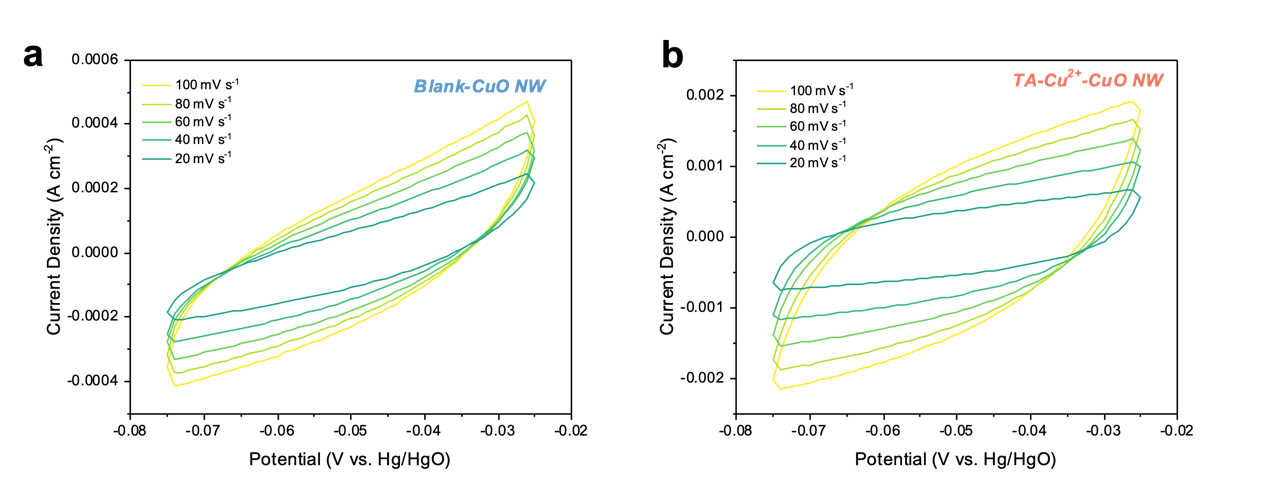


**Figure S10.** CV curves of a) Blank-CuO NW/Cu foam and b) TA-Cu^2+^-CuO NW/Cu foam at scan rate of 20 mV s^-1^ to 100 mV s^-1^.

**Figure S11.** Electrochemical double-layer capacity (C_dl_) of Blank-CuO NW/Cu foam and TA-Cu^2+^-CuO NW/Cu foam.

**Figure S12.** Comparison of long-term stability test for TA-Cu^2+^-CuO NW/Cu foam and blank-CuO NW/Cu foam in three-electrode H-cell under acidic condition.


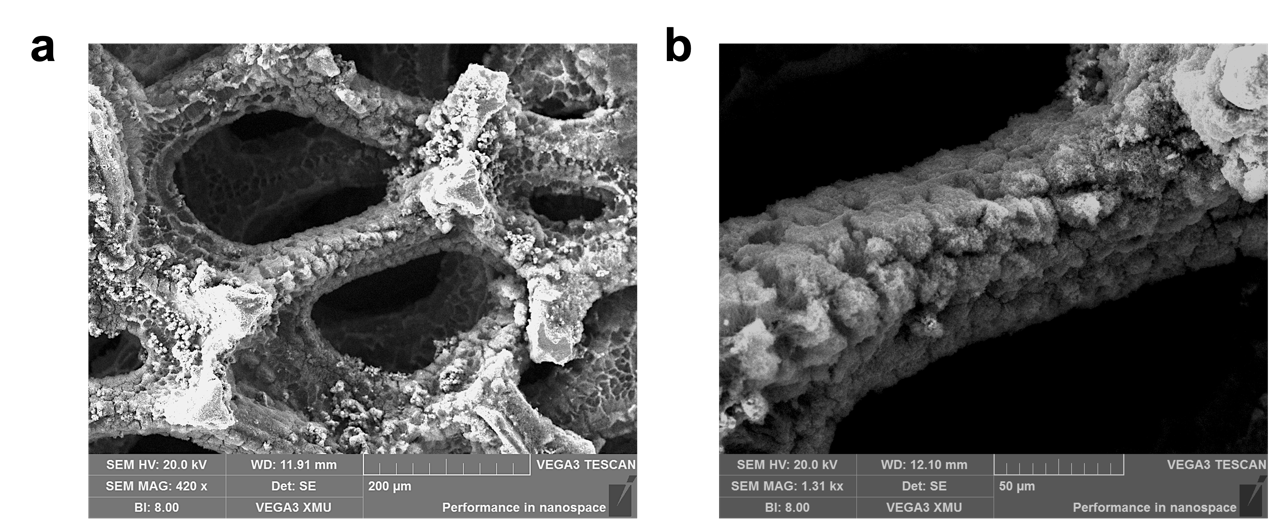


**Figure S13.** SEM image of blank-CuO NW/Cu foam after 20 min operation.

**
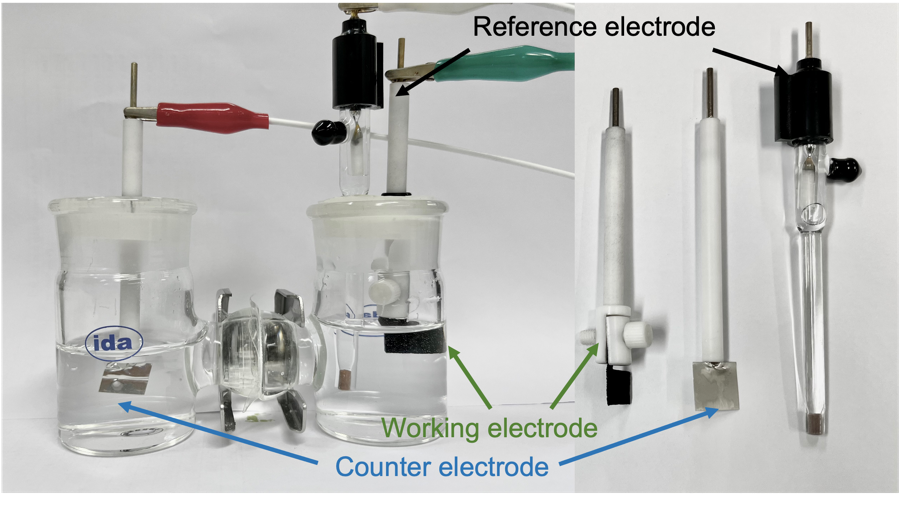
**

**Figure S14.** Schematic of H-cell reactor (three-electrode system).


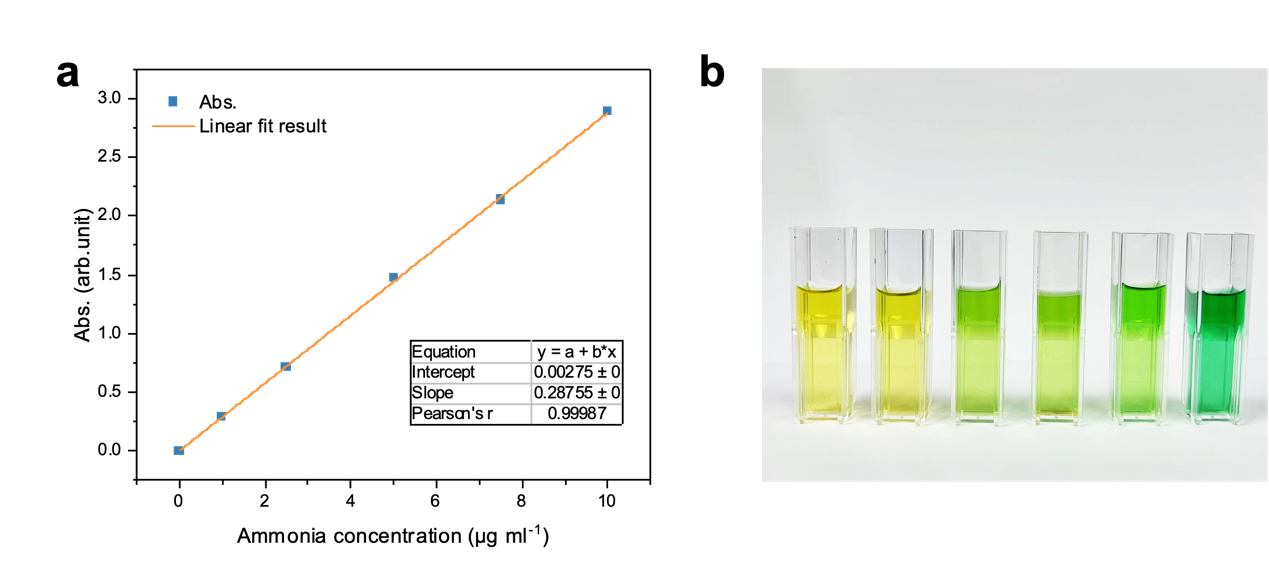


**Figure S15.** a) Linear fitting of the corresponding calibration curve for the relationship between the absorbance and ammonia concentration. b) Photograph of solution colored by the indophenol indicator with increasing ammonia concentration.


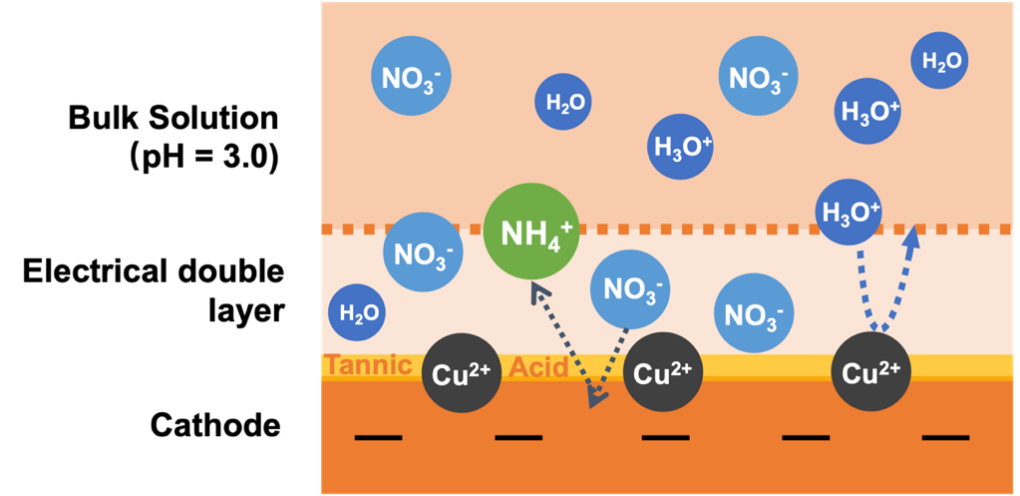


**Figure S16.** Schematic of proposed IEF mechanism for nitrate ions and hydronium ions in acidic condition (pH = 3.0).

**Figure S17.** Comparison of the RDE LSV curves of HER between TA-Cu^2+^-CuO NW/Cu foam and Blank-CuO NW/Cu foam in 0.5 M K_2_SO_4_.

**Figure S18.** Comparison of the LSV curves between TA-Cu^2+^-CuO NW/Cu foam and Blank-CuO NW/Cu foam of NO_3_RR in acidic solution (0.5 M K_2_SO_4_ + H_2_SO_4_, pH =3.0).


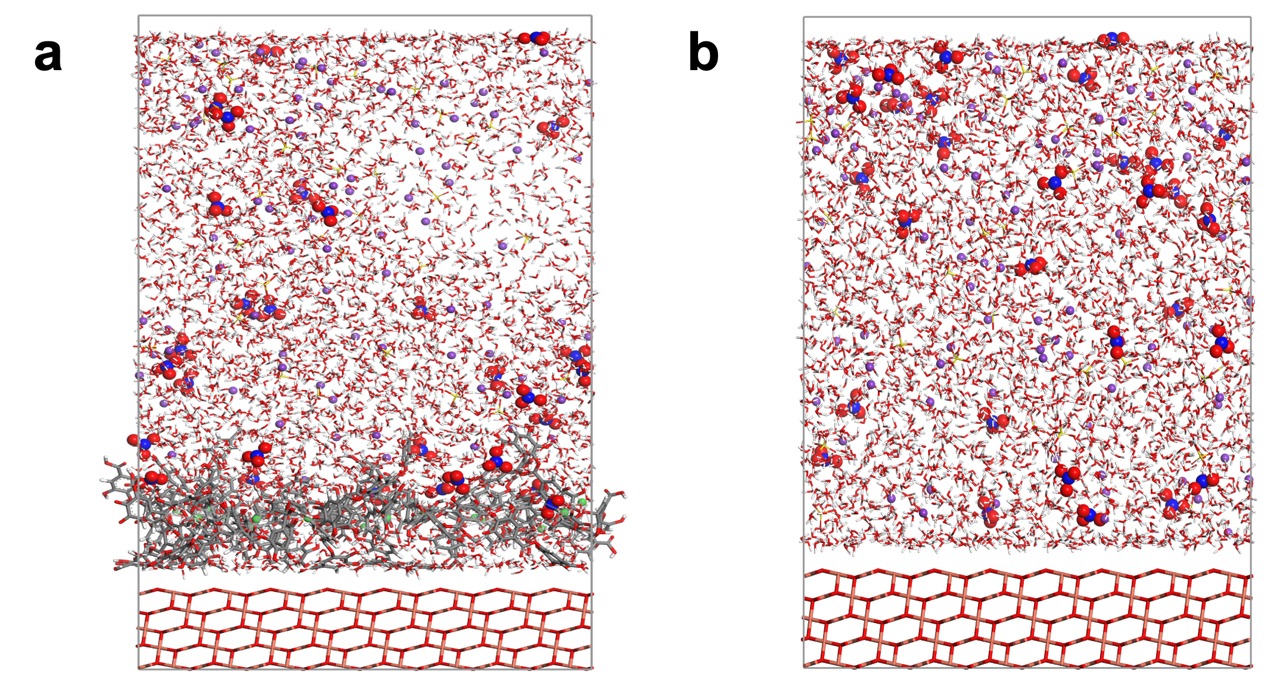


**Figure S19.** MD simulation model of (a) CuO (111) plane with TA-Cu^2+^ and (b) blank CuO (111) plane in 0.5 M K_2_SO_4_ + 2000 ppm KNO_3_ solution.

**Figure S20.** Number of potassium ions (K^+^) along the z-axis from the electrode surface of TA-Cu^2+^-CuO and Blank-CuO surfaces based on MD simulations.

**Figure S21.** LSV curves of commercial Cu foam and TA-treated Cu foam in the electrolyte of 0.5 M K_2_SO_4_ and 2000 KNO_3_ of three-electrode H-cell.

**Figure S22.** LSV curves of commercial Cu foil and TA-treated Cu foil in the electrolyte of 0.5 M K_2_SO_4_ and 2000 KNO_3_ of three-electrode H-cell.


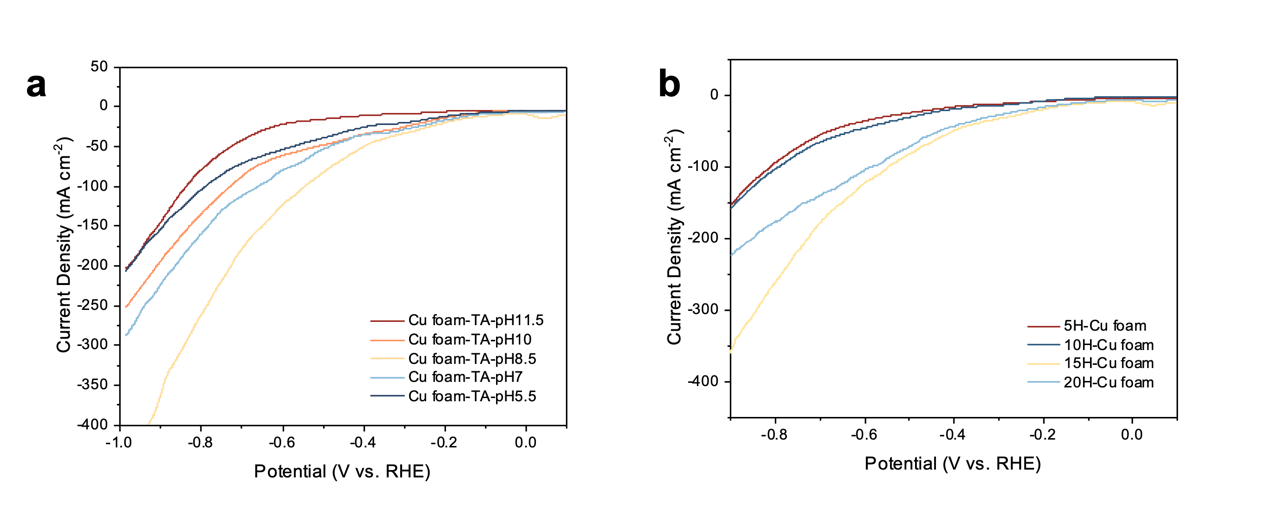


**Figure S23.** LSV curves of TA-treated Cu foam with different synthesis conditions of a) pH value and b) reaction time (Electrolyte: 0.5 M K_2_SO_4_ and 2000 KNO_3_).


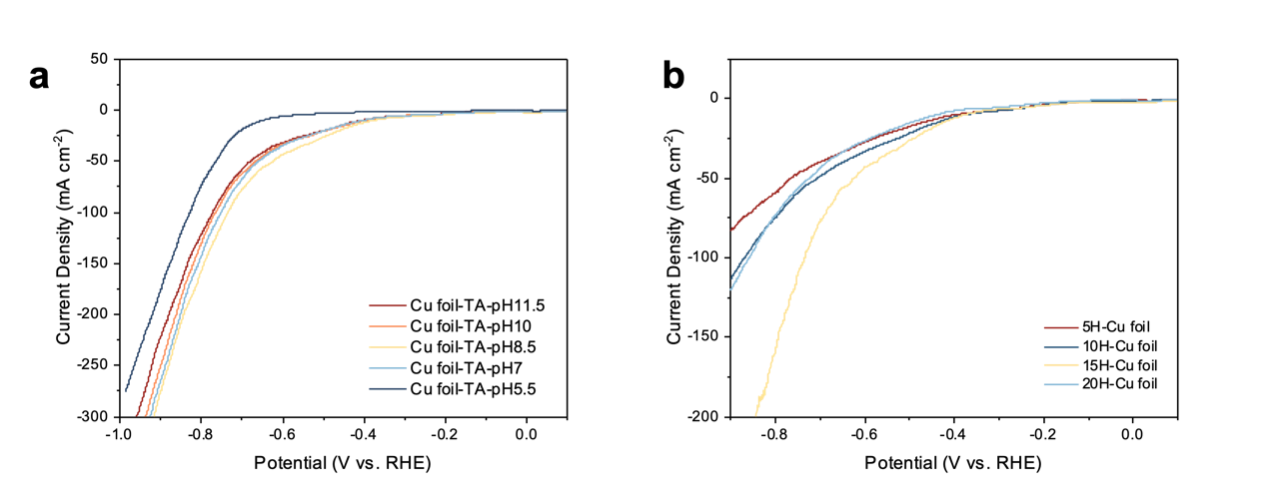


**Figure S24.** LSV curves of TA-treated Cu foil with different synthesis conditions of a) pH value and b) reaction time (Electrolyte: 0.5 M K_2_SO_4_ and 2000 KNO_3_).


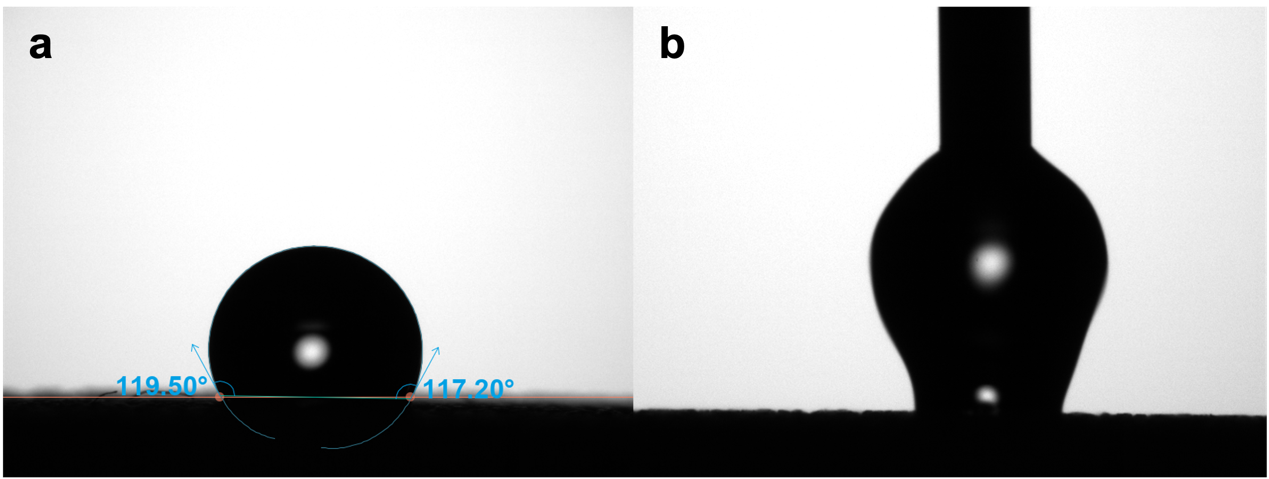


**Figure S25.** Contact angle of a) commercial Cu foam and b) TA-treated Cu foam.

**Figure S26.** Faraday efficiency comparison of commercial Cu foam with/without TA treatment in 0.5 M K_2_SO_4_ and 2000 KNO_3_.


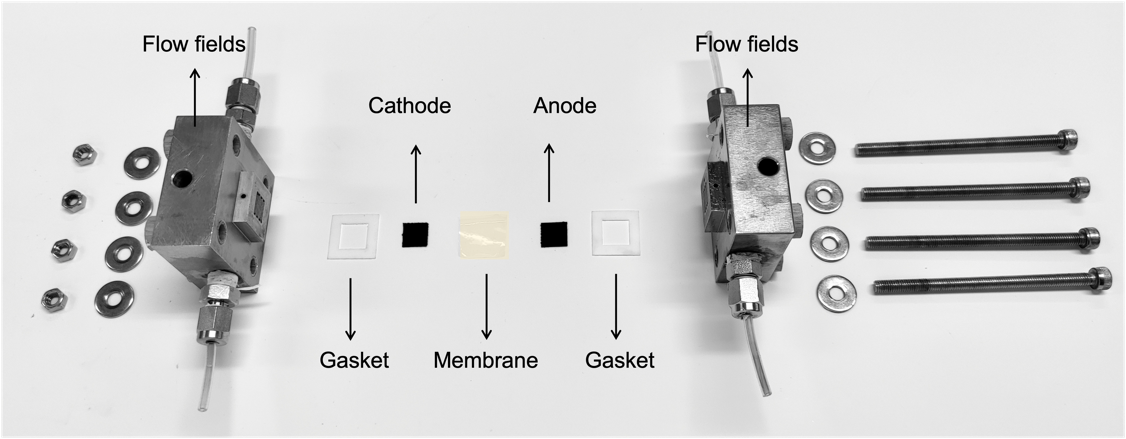


**Figure S27.** Schematic of the flow cell components (active area: 1 cm × 1 cm).


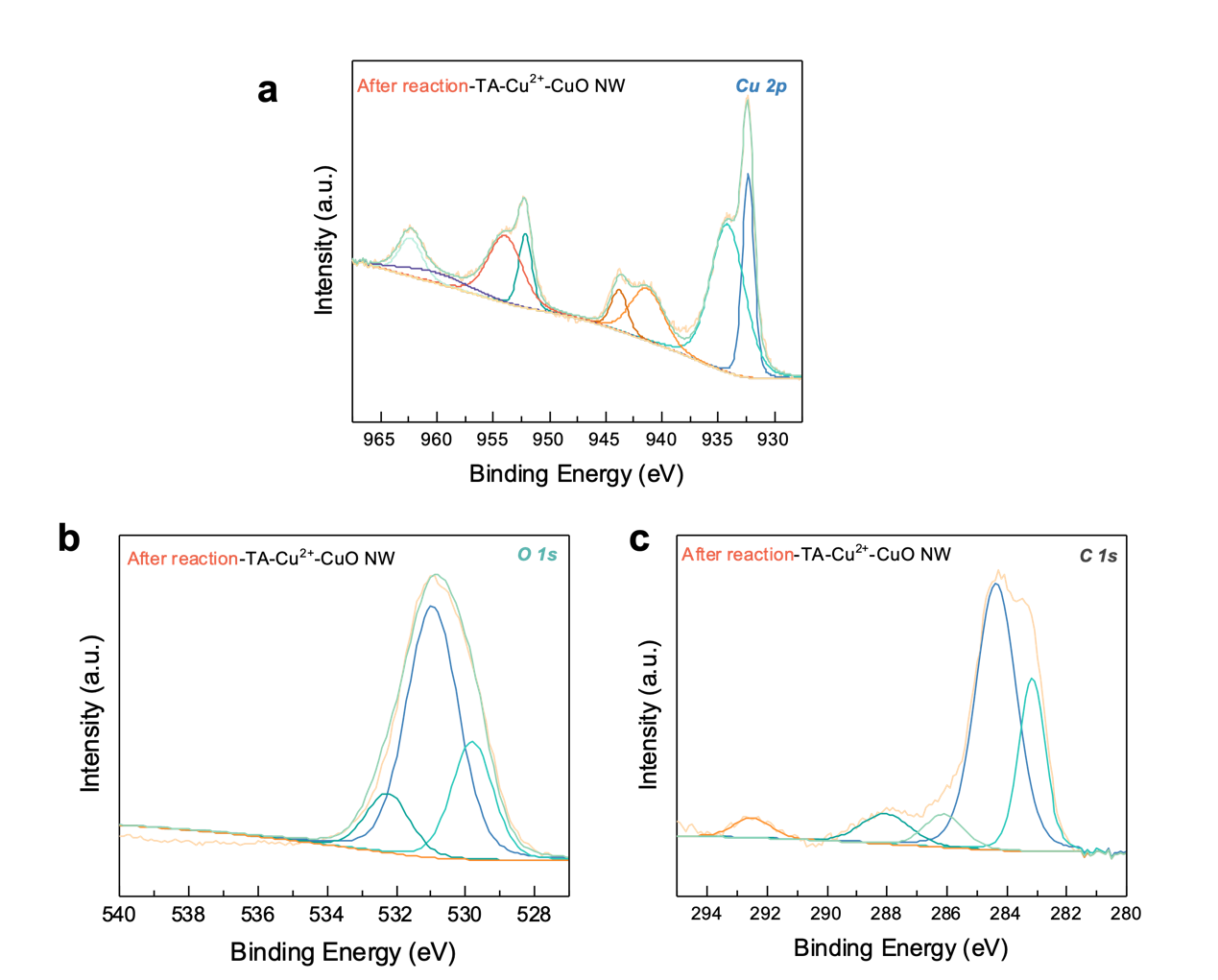


**Figure S28.** XPS spectra of a) Cu 2p, b) O 1s and c) C 1s of TA-Cu^2+^-CuO NW/Cu foam after the long-term stability test.


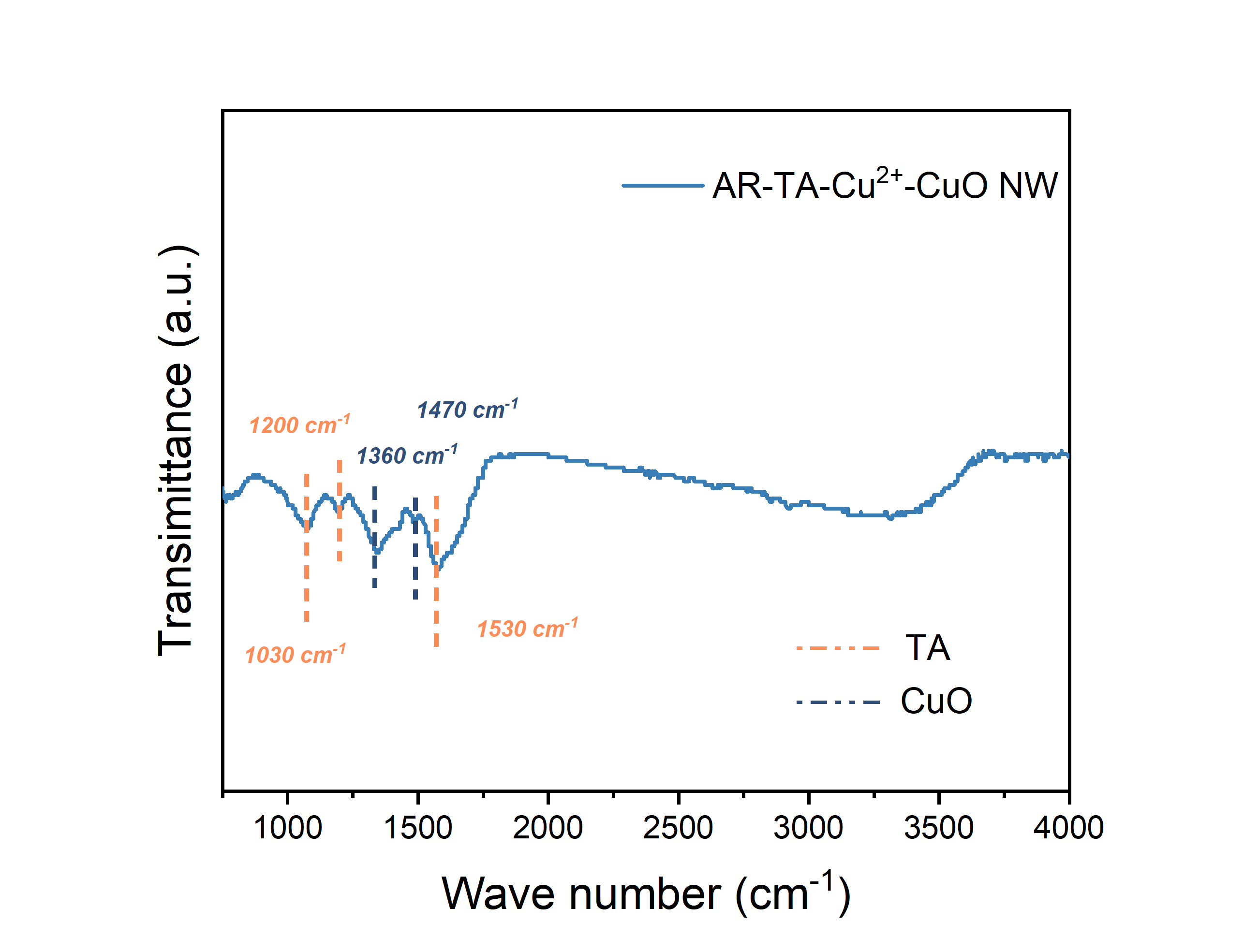


**Figure S29.** FTIR spectra of TA-Cu^2+^-CuO NW/Cu foam after the long-term stability test.

*
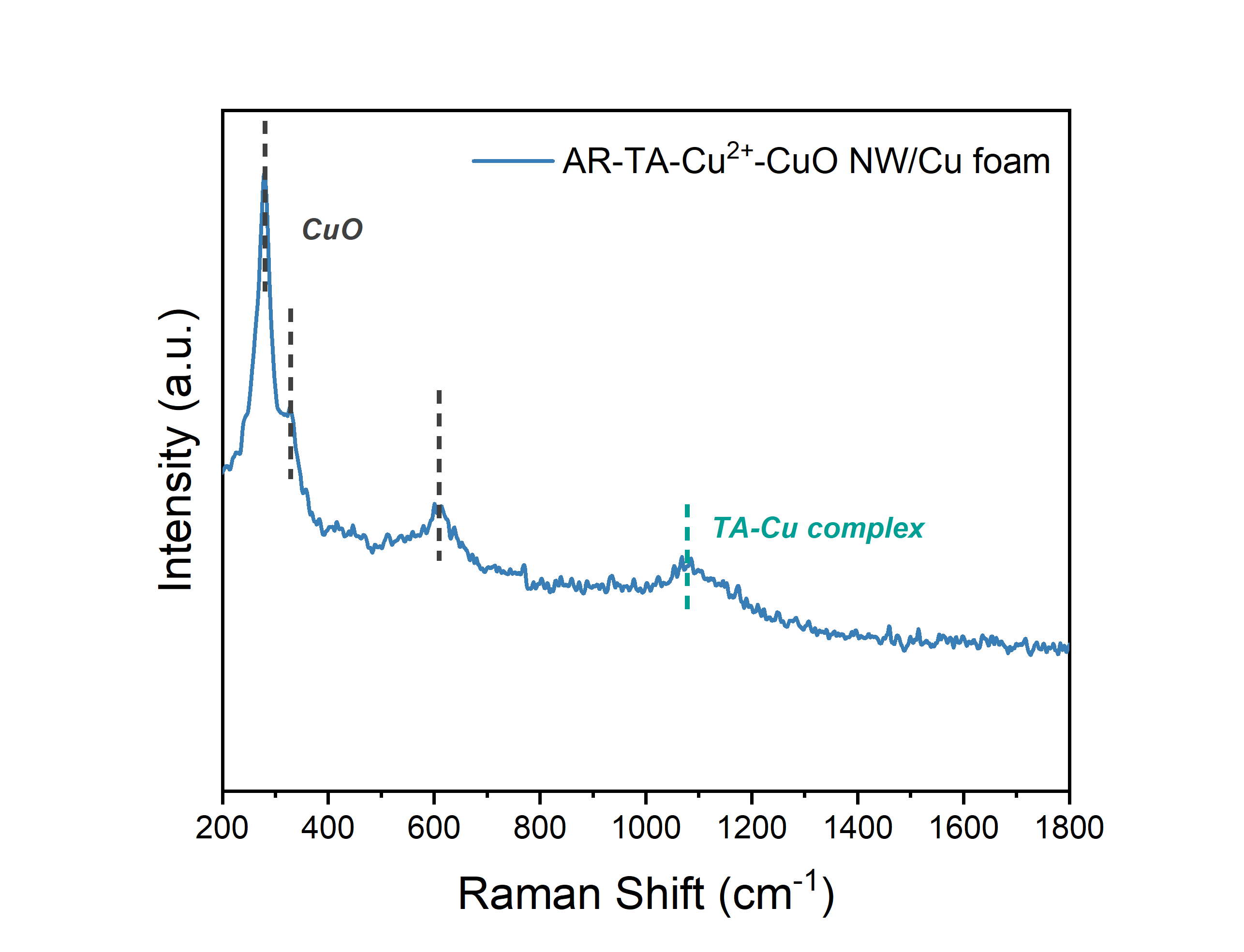
*

**Figure S30.** Raman spectra of TA-Cu^2+^-CuO NW/Cu foam after the long-term stability test.

*
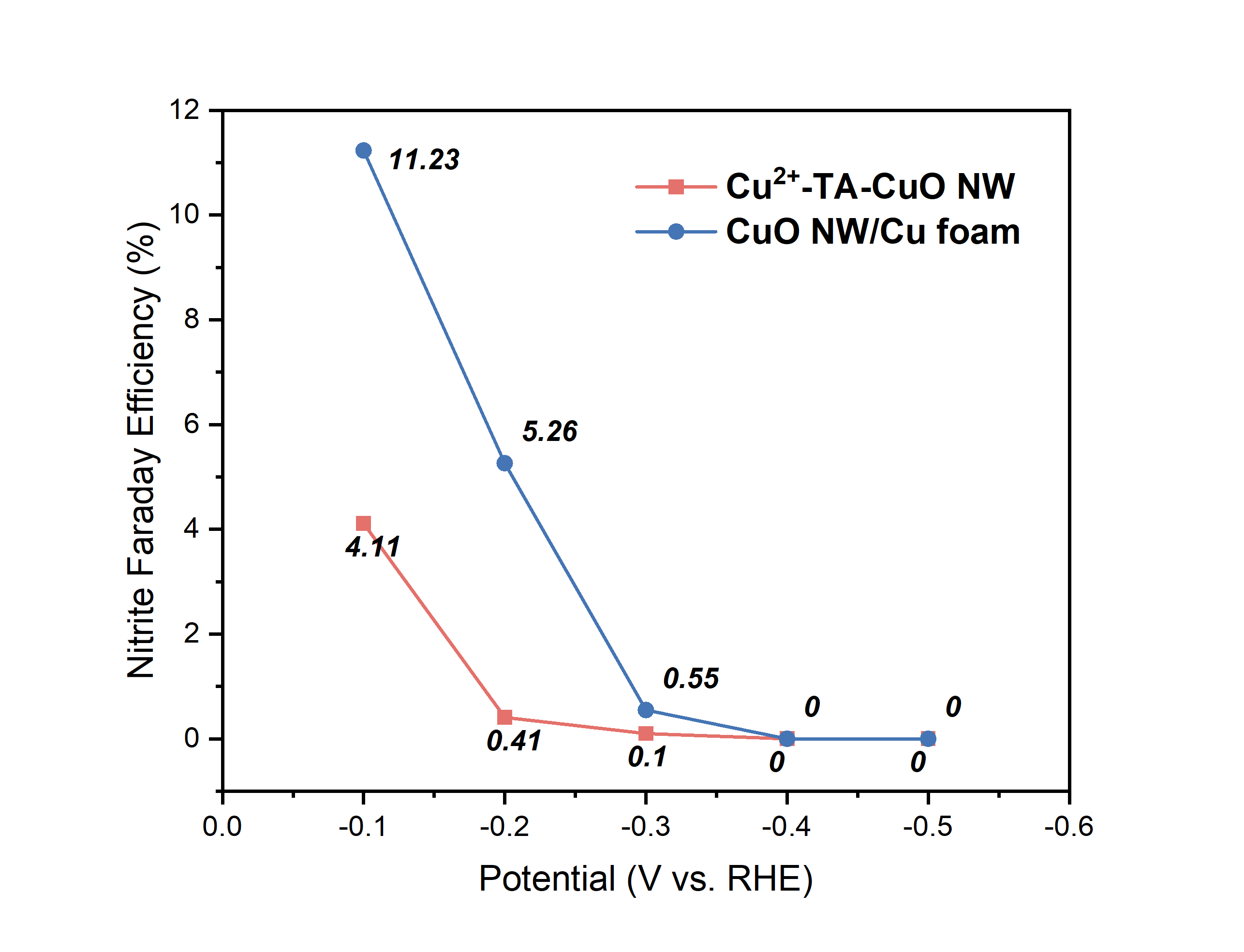
*

**Figure S31.** Nitrite Faraday efficiency under various potentials in the electrolyte of 0.5 M K_2_SO_4_ and 2000 ppm KNO_3_.


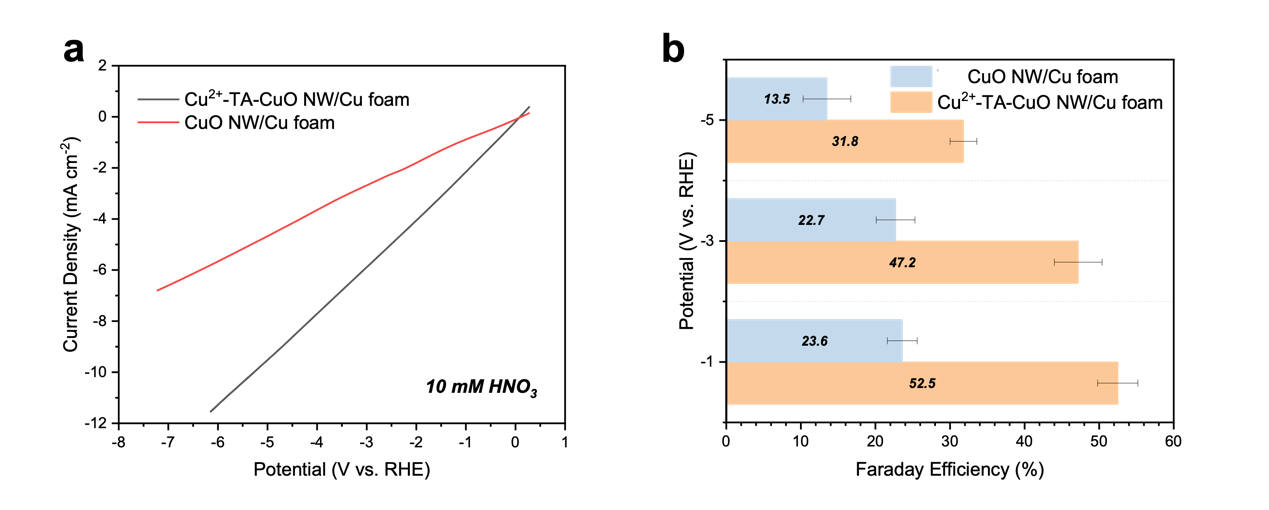


**Figure S32.** a) LSV curves and b) ammonia FE under various potentials of CuO NW/Cu foam and TA-Cu^2+^-CuO NW/Cu foam in the electrolyte of 10 mM HNO_3_.


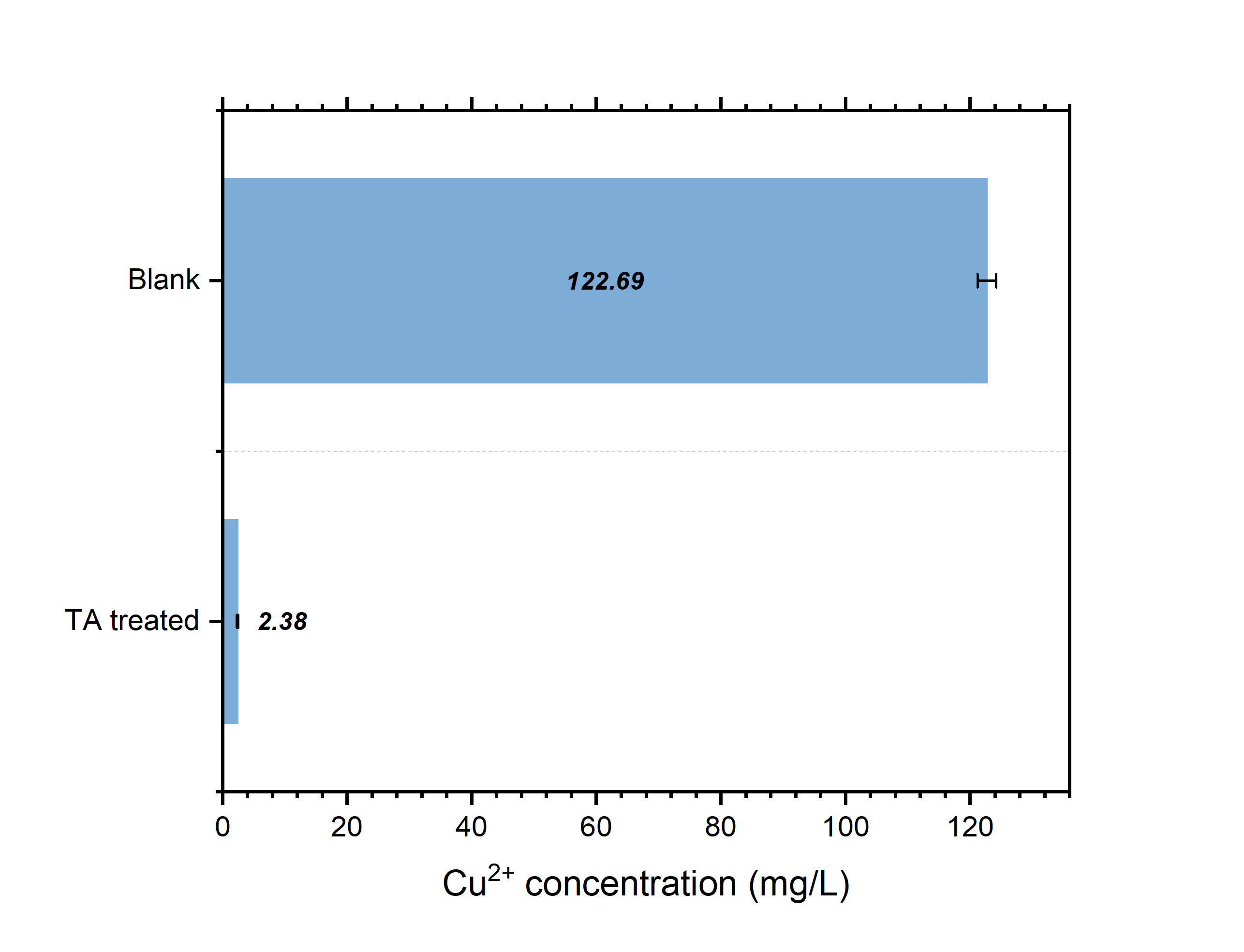


**Figure S33.** Comparison of the Cu^2+^ concentration in the electrolyte after 1 h continuous reaction in acidic electrolyte (pH =3).


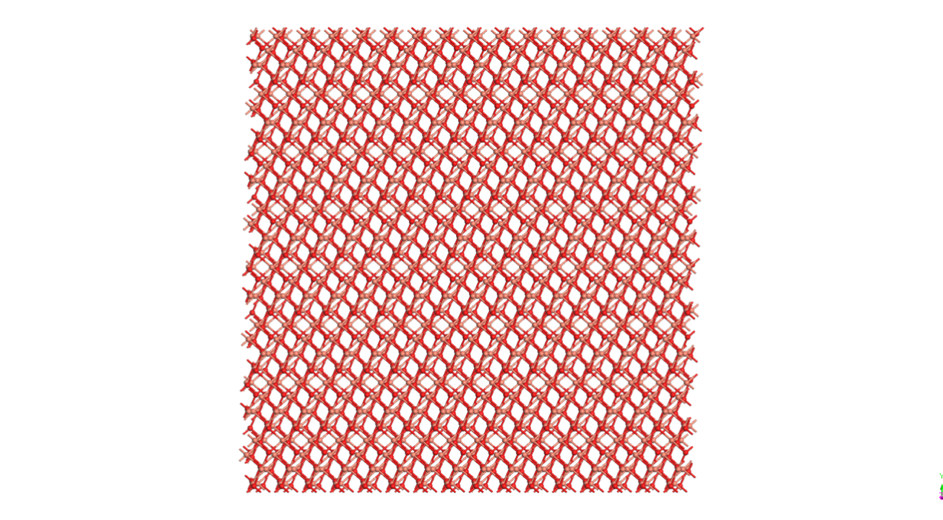


**Figure S34.** Model of CuO (111) plane for MD simulation.


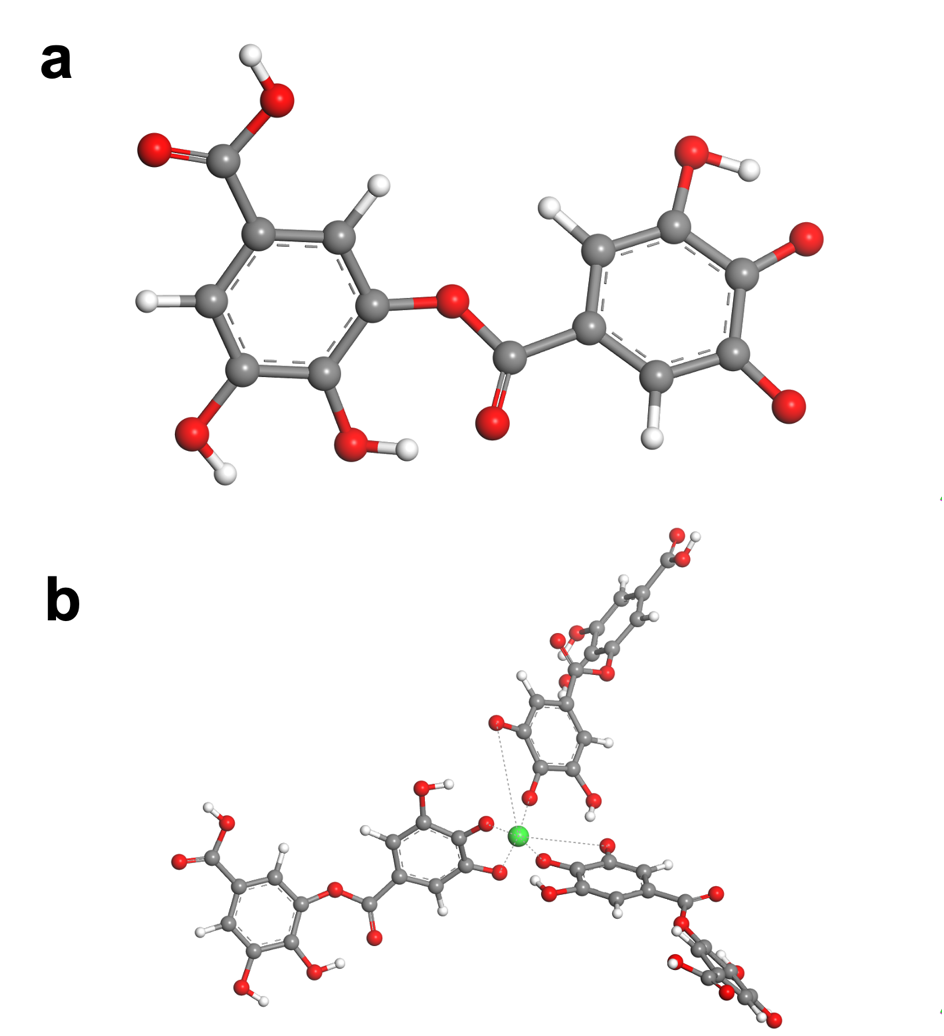


**Figure S35.** Simplified model of a) TA and b) TA-Cu^2+^ for MD simulation.

**Table S1.** Element component of TA-Fe^2+^-CuO NW/Cu foam.

| Element | Weight% | Atomic% |
| --- | --- | --- |
| C K | 12.96 | 29.50 |
| O K | 25.82 | 44.14 |
| Fe K | 0.28 | 0.14 |
| Cu K | 60.94 | 26.22 |
| Totals | 100.00 | 100.00 |

**Table S2.** Element component of TA-Co^2+^-CuO NW/Cu foam.

| Element | Weight% | Atomic% |
| --- | --- | --- |
| C K | 16.55 | 33.40 |
| O K | 30.57 | 46.32 |
| Co K | 0.31 | 0.23 |
| Cu K | 52.57 | 20.05 |
| Totals | 100.00 | 100.00 |

**Table S3.** Element component of TA-Cu^2+^-CuO NW/Cu foam.

| Element | Weight% | Atomic% |
| --- | --- | --- |
| C K | 16.60 | 33.48 |
| O K | 30.67 | 46.43 |
| Cu K | 52.73 | 20.09 |
| Totals | 100.00 | 100.00 |

**Table S4.** Cu ionic component of TA-Cu^2+^-CuO NW/Cu foam (S1) and CuO/Cu foam (S2) of electrolyte after 10 hours acidic operation (initial pH = 3) through ICP test.

| Sample number | Element | Tested sample C_o_ (mg/L) | Dilution times f | Original sample C_1_ (mg/L) |
| --- | --- | --- | --- | --- |
| S1 | Cu | 0.0475 | 50 | 2.3737 |
| S1 | Cu | 0.0474 | 50 | 2.3705 |
| S1 | Cu | 0.0482 | 50 | 2.4113 |
| S2 | Cu | 2.4398 | 50 | 121.9895 |
| S2 | Cu | 2.4827 | 50 | 124.1365 |
| S2 | Cu | 2.4388 | 50 | 121.9400 |

**References:**

1. H. Sun, P. Ren, J. Fried, The COMPASS force field: parameterization and validation for phosphazenes. *Computational and Theoretical Polymer Science* **8**, 229-246 (1998).

2. H. Sun, COMPASS: an ab initio force-field optimized for condensed-phase applications overview with details on alkane and benzene compounds. *The Journal of Physical Chemistry B* **102**, 7338-7364 (1998).

3. H. Jürgen, Ab-initio simulations of materials using VASP: Density-functional theory and beyond. *Journal of Computational Chemistry* **29**, 2044-2078 (2008).

4. Blöchl, Projector augmented-wave method. *Physical Review. B, Condensed Matter* **50**, 17953-17979 (1994).

5. Perdew, Burke, Wang, Generalized gradient approximation for the exchange-correlation hole of a many-electron system. *Physical Review. B, Condensed Matter* **54**, 16533-16539 (1996).

6. G. Stefan, Semiempirical GGA-type density functional constructed with a long-range dispersion correction. *Journal of Computational Chemistry* **27**, 1787-1799 (2006).

7. J.-Y. Fang *et al.*, Ampere-level current density ammonia electrochemical synthesis using CuCo nanosheets simulating nitrite reductase bifunctional nature. *Nature Communications* **13**, 7899 (2022).

8. J. K. Norskov *et al.*, Trends in the exchange current for hydrogen evolution. *Journal of the Electrochemical Society* **152**, J23-J26 (2005).

9. S. Guo *et al.*, Insights into Nitrate Reduction over Indium-Decorated Palladium Nanoparticle Catalysts. *ACS Catalysis* **8**, 503-515 (2018).

10. T. Hu, C. Wang, M. Wang, C. M. Li, C. Guo, Theoretical Insights into Superior Nitrate Reduction to Ammonia Performance of Copper Catalysts. *ACS Catalysis* **11**, 14417-14427 (2021).

11. R. Tomaszewski, Citations to chemical resources in scholarly articles: CRC Handbook of Chemistry and Physics and The Merck Index. *Scientometrics* **112**, 1865-1879 (2017).
